# Nonclassical action of Ku70 promotes Treg-suppressive function through a FOXP3-dependent mechanism in lung adenocarcinoma

**DOI:** 10.1172/JCI178079

**Published:** 2024-10-24

**Authors:** Qianru Huang, Na Tian, Jianfeng Zhang, Shiyang Song, Hao Cheng, Xinnan Liu, Wenle Zhang, Youqiong Ye, Yanhua Du, Xueyu Dai, Rui Liang, Dan Li, Sheng-Ming Dai, Chuan Wang, Zhi Chen, Qianjun Zhou, Bin Li

**Affiliations:** 1 Center for Immune-Related Diseases at Shanghai Institute of Immunology, Department of Respiratory and Critical Care Medicine and Department of Thoracic Surgery of Ruijin Hospital, Department of Immunology and Microbiology, Shanghai Jiao Tong University School of Medicine, Shanghai, China.; 2 Department of Rheumatology and Immunology, Shanghai Sixth People’s Hospital Affiliated to Shanghai Jiao Tong University School of Medicine, Shanghai, China.; 3 Department of Thoracic Surgery, Shanghai Chest Hospital, Shanghai Jiao Tong University, Shanghai, China.; 4 Department of Thoracic Surgery, Renji Hospital, Shanghai Jiao Tong University School of Medicine, Shanghai, China.; 5 Department of Rheumatism and Immunology, Peking University Shenzhen Hospital, Guangdong, China.; 6 Department of Obstetrics and Gynecology, Renji Hospital, Shanghai Jiao Tong University School of Medicine, Shanghai, China.; 7 Intercollegiate Faculty of Biotechnology of University of Gdańsk and Medical University of Gdańsk, University of Gdańsk, Gdańsk, Poland.; 8 Faculty of Biochemistry and Molecular Medicine, University of Oulu, Oulu, Finland.; 9 Department of Integrated TCM & Western Medicine, Shanghai Skin Disease Hospital; Department of Thoracic Surgery, Shanghai Pulmonary Hospital, Tongji University School of Medicine, Shanghai, China.; 10 Shanghai Guanghua Hospital of Integrative Medicine, Shanghai University of Traditional Chinese Medicine, Shanghai, China.; 11 Department of Oncology, Department of Hepatobiliary Surgery, The First Affiliated Hospital of Anhui Medical University, Hefei, China.

**Keywords:** Immunology, Oncology, Lung cancer, T cells

## Abstract

Ku70, a DNA repair protein, binds to the damaged DNA ends and orchestrates the recruitment of other proteins to facilitate repair of DNA double-strand breaks. Besides its essential role in DNA repair, several studies have highlighted nonclassical functions of Ku70 in cellular processes. However, its function in immune homeostasis and antitumor immunity remains unknown. Here, we discovered a marked association between elevated Ku70 expression and unfavorable prognosis in lung adenocarcinoma, focusing specifically on increased Ku70 levels in tumor-infiltrated Tregs. Using a lung-colonizing tumor model in mice with Treg-specific Ku70 deficiency, we demonstrated that deletion of Ku70 in Tregs led to a stronger antitumor response and slower tumor growth due to impaired immune-suppressive capacity of Tregs. Furthermore, we confirmed that Ku70 played a critical role in sustaining the suppressive function of human Tregs. We found that Ku70 bound to forkhead box protein P3 (FOXP3) and occupied FOXP3-bound genomic sites to support its transcriptional activities. These findings not only unveil a nonhomologous end joining–independent (NHEJ-independent) role of Ku70 crucial for Treg-suppressive function, but also underscore the potential of targeting Ku70 as an effective strategy in cancer therapy, aiming to both restrain cancer cells and enhance pulmonary antitumor immunity.

## Abstract 2

Our research delves into the NHEJ-independent role of Ku70 in the regulation of regulatory T (Treg) cells, which impacts pulmonary anti-tumor immune responses.

## Introduction

Ku70, encoded by *XRCC6*, is well known as forming a heterodimer with Ku80 participating in repairing DNA double-strand breaks (DSBs) by nonhomologous end joining (NHEJ) (1). Many studies have highlighted that cancer cells with Ku70 deletion were genetically unstable and hypersensitive to therapeutic agents that caused DNA damage, such as chemotherapy and radiotherapy (2, 3). Moreover, the absence of Ku70 disrupts NHEJ, impacting V(D)J recombination and causing impaired lymphocyte development, ultimately resulting in SCID in mice (4). Ku70 also exhibits heterodimer-independent and non-DNA repair functions. In innate immunity, Ku70 serves as a cytosolic DNA sensor to induce interferons and proinflammatory cytokines (5). Additionally, Ku70 also detects cytoplasmic DNA in aged CD4^+^ T cells, promoting T cell activation and potentiating aging-related autoimmunity (6). However, it remains unclear whether Ku70 plays novel roles in diverse cells of adaptive immunity.

Tregs, a distinctive subset of CD4^+^ T cells endowed with immunosuppressive capabilities, play a pivotal role in maintaining immune tolerance while simultaneously hindering antitumor immunity (7). The immune-suppressive mechanisms employed by Tregs encompass the inhibition of costimulatory signals by cytotoxic T lymphocyte antigen-4 (CTLA4), the deprivation of IL-2 by CD25, and the secretion of antiinflammatory cytokines, such as IL-10, among other strategies (8). Categorized by their maturation sites, Tregs are classified into 3 clusters: thymus-derived Tregs (tTregs), peripherally derived Tregs (pTregs), and in vitro–induced Tregs (iTregs) (9). The master transcription factor forkhead box protein P3 (FOXP3) governs the development and function of Tregs under diverse conditions. The stability and binding partners of FOXP3 regulate the plasticity of Treg function. Previous reports demonstrated some factors, such as Neuropilin-1 (NRP1) (10) and enhancer of zeste homolog 2 (EZH2) (11), restrictively deleted in Tregs impaired the transcription activity of FOXP3, restricting Treg-suppressive ability and conferring resistance to tumor growth in the host. As increasing data illuminate the intricate roles of Tregs in tumor, the strategic targeting of Tregs emerges as a promising approach for tumor therapy.

Lung adenocarcinoma (LUAD) is the predominant subtype of lung cancer, and patients with LUAD can benefit from various treatment options, including surgery, chemotherapy, radiotherapy, targeted therapy, and immunotherapy (12). However, further research is still urgently needed to integrate multiple treatment strategies and improve therapeutic effects. Here, we unearthed a negative correlation between Ku70 expression and the prognosis of LUAD and highlighted the elevated Ku70 levels in tumor-infiltrating Tregs. Specific knockout of Ku70 in Tregs impaired the Treg-suppressive function and enhanced the antitumor response to limit the development of lung-colonizing tumors. And the same changes could be detected in human Tregs. Furthermore, we uncovered that Ku70 sustained the suppressive ability of Tregs in LUAD by interacting with FOXP3 to strengthen the transcriptional activity of FOXP3.

## Results

### The expression of Ku70 is negatively correlated with prognosis of LUAD with an increased level in tumor-infiltrating Tregs.

**Figure 1:**
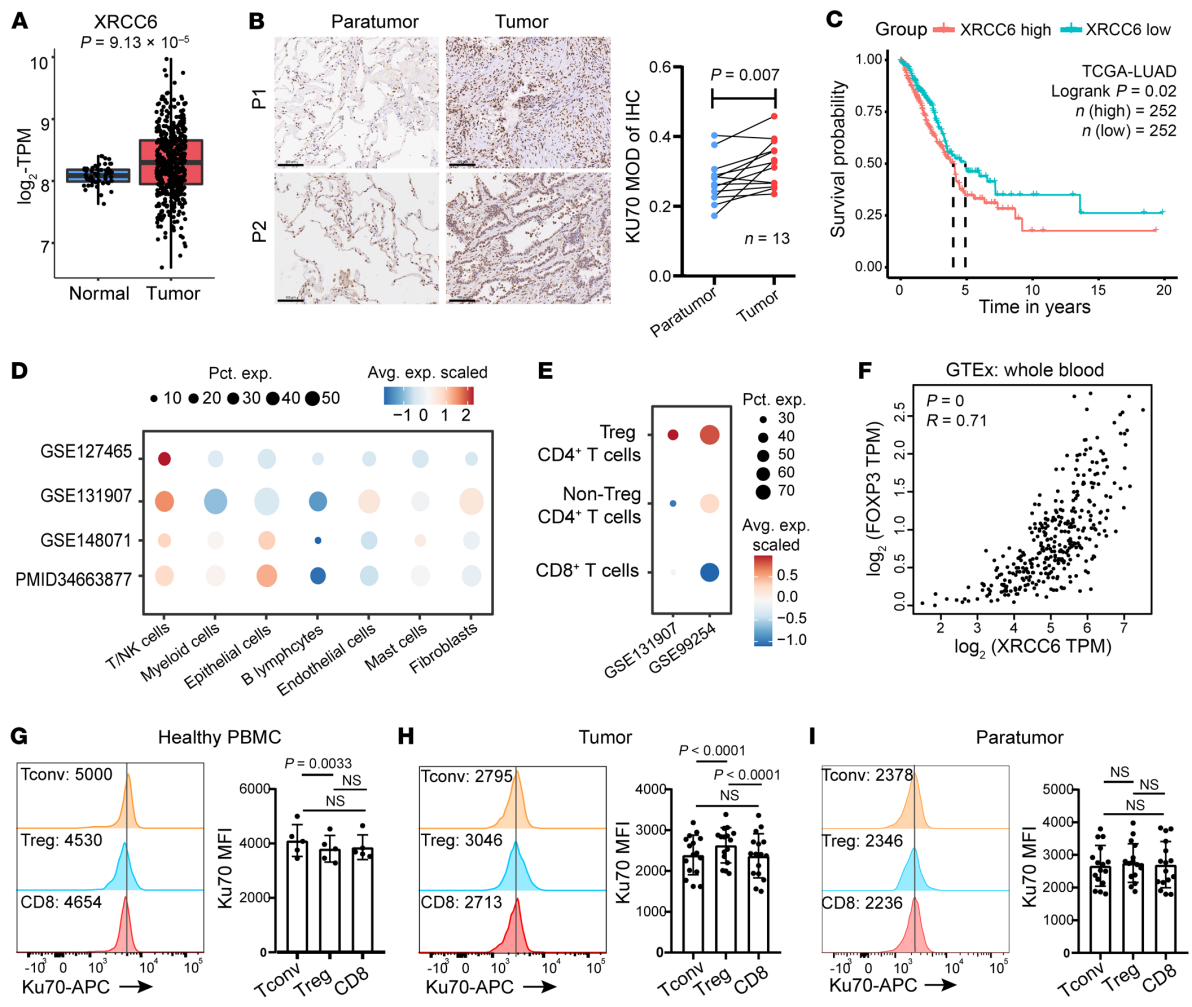
Increased Ku70 expression indicates an adverse prognosis in LUAD, particularly enhanced in tumor-infiltrating Tregs. (**A**) The expression of *XRCC6* between normal (*n* = 58) and LUAD tissues (*n* = 513) from the TCGA database. (**B**) Immunohistochemical analysis of Ku70 expression in tissues from LUAD patients (*n* = 13). Scale bar: 100 μm. (**C**) Overall survival analysis based on *XRCC6* gene expression (median) using Kaplan–Meier curves in the TCGA-LUAD cohort. The red line represents patients with high *XRCC6* expression, while the blue line represents those with low expression. *P* values were calculated using log-rank test. (**D** and **E**) Dot plot depicting *XRCC6* expression in different cell clusters. The dot size reflected the percentage of cells in a cluster expressing each gene. The dot color reflected the expression level. (**F**) Correlation between *FOXP3* expression and *XRCC6* expression in whole blood from GTEx database. Spearman’s correlation was used for calculation. (**G**–**I**) Quantification of Ku70 expression in PBMCs from healthy donors (*n* = 5; **G**), tumor tissues (**H**), and paratumor tissues (**I**) from LUAD patients (*n* = 16). Statistical analyses were performed using Student’s paired *t* test between Tregs and Tconv cells from donors and Tregs and CD8^+^ cells as well as between CD8^+^ and Tconv cells. Data are represented as means ± SD.

To assess Ku70 levels in lung cancer, we utilized RNA-Seq data from the The Cancer Genome Atlas–LUAD (TCGA-LUAD) dataset. Our analysis unveiled heightened *XRCC6* expression in LUAD tumors compared with adjacent normal tissues (Figure 1A). Immunohistochemical analysis further confirmed markedly elevated levels of Ku70 protein staining in LUAD tissues compared with adjacent nontumor tissues (Figure 1B). Moreover, we investigated the correlation between *XRCC6* expression and prognosis in cancer patients. *XRCC6* overexpression was associated with an unfavorable prognosis in LUAD (Figure 1C). Interestingly, its expression showed no correlation with prognosis in lung squamous cell carcinoma (LUSC), colon cancer (COAD), and liver cancer (LIHC) (Supplemental Figure 1A; supplemental material available online with this article; https://doi.org/10.1172/JCI178079DS1), suggesting that the prognostic importance of Ku70 expression is influenced by the unique tumor microenvironment (TME) of LUAD.

Previous studies primarily emphasized the impact of Ku70 in cancer cells on sensitivity to radiotherapy and chemotherapy (2, 13). Using the transcriptome of LUAD from 4 public single-cell RNA-Seq data sets (14–17), we unraveled the heterogeneity of *XRCC6* expression across different cell types. Strikingly, *XRCC6* exhibited high expression in T/NK cells of LUAD cancer (Figure 1D). Further, 2 public data sets were employed to discern the differential expression of *XRCC6* in various clusters of T cells in LUAD (15, 18). They revealed elevated *XRCC6* expression in Treg CD4^+^ T cells compared with non-Treg CD4^+^ T cells and CD8^+^ T cells in LUAD cancer (Figure 1E). Correlation analysis showed a positive relationship between *XRCC6* expression and *FOXP3* expression in whole blood data from the GTEx database (https://gtexportal.org/home/) (Figure 1F).

Subsequently, we collected paired tumor tissues and adjacent normal tissues from patients with LUAD before the treatment (Supplemental Tables 1 and 2) as well as PBMCs from healthy donors to analyze Ku70 expression. The results showed that Tregs in healthy PBMCs expressed a lower level of Ku70 compared with conventional CD4^+^ T (Tconv) cells (Figure 1G). In contrast, Tregs in LUAD exhibited higher Ku70 expression compared with Tconv and CD8^+^ T cells (Figure 1H). Concurrently, Ku70 expression showed no substantial difference in different clusters of adjacent normal tissue (Figure 1I). This suggests that tumor-infiltrated Tregs express high levels of Ku70 in LUAD tissue, potentially contributing to tumor progression. In summary, these findings underscore the negative correlation of Ku70 expression with the prognosis of LUAD and its upregulation in tumor-infiltrated Tregs.

### FOXP3 conditional knockout of Ku70 in mice results in heightened T cell activation and a reduced proportion of tissue Tregs.

**Figure 2:**
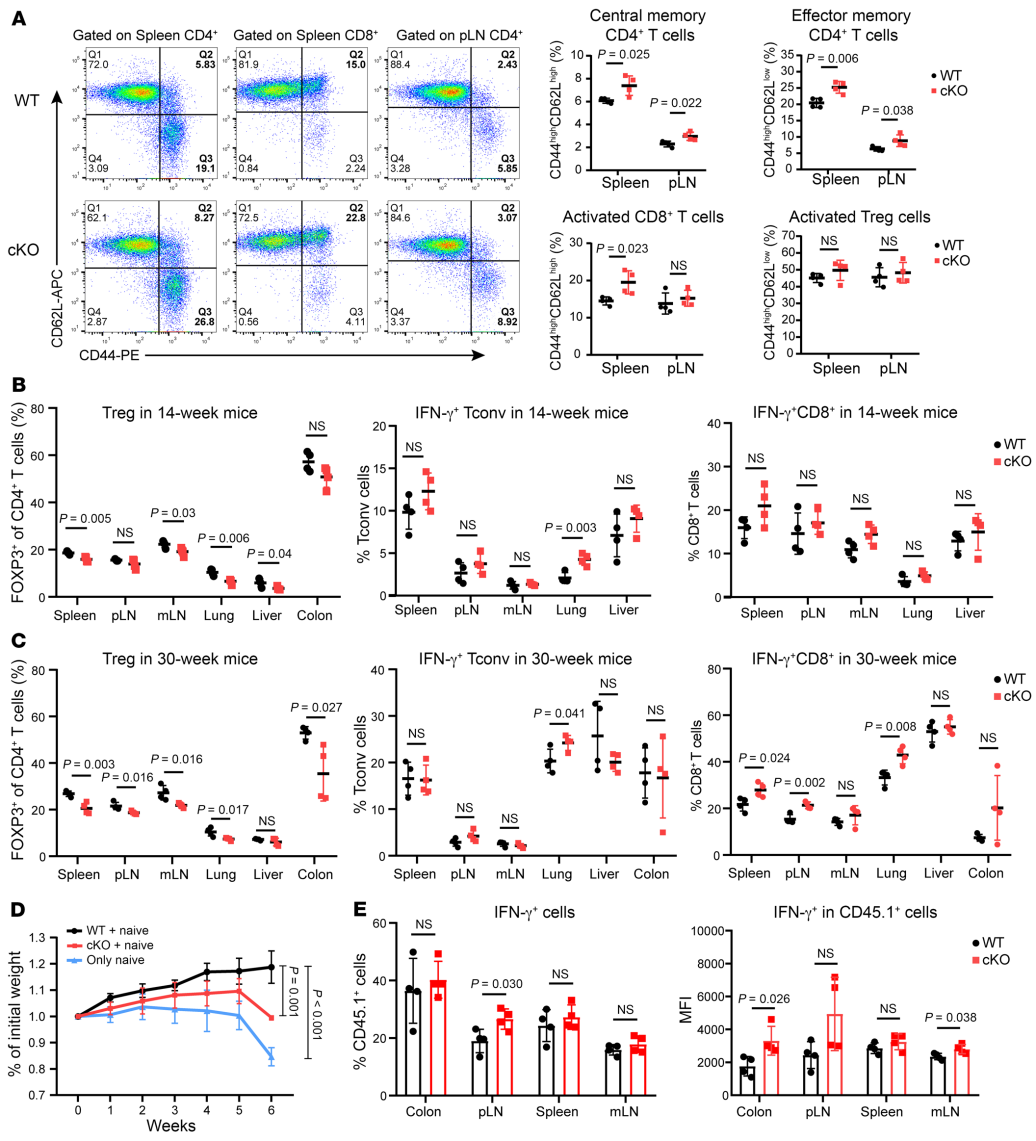
Treg-specific deletion of Ku70 enhances T cell activation in spleen and pLN. (**A**) Representative flow cytometry staining (left) for CD62L and CD44 on the surface of different subpopulations of T cells in spleen and pLNs of WT and cKO mice (14 weeks old, *n* = 4) and summaries (right) for the frequencies of the activation status of T cells are shown. (**B**) Frequency of Tregs and IFN-γ–producing CD4^+^ and CD8^+^ T cells in different organs in WT and cKO mice (14 weeks old, *n* = 4). (**C**) Frequency of Tregs and IFN-γ–producing CD4^+^ and CD8^+^ T cells in different organs in WT and cKO mice (30 weeks old, *n* = 4). (**D** and **E**) Tregs were sorted from the spleens of WT and cKO mice. Rag^–/–^ mice (*n* = 4) were injected with 0.45 × 10^6^ naive CD4^+^CD45.1^+^ T cells alone or coinjected with 0.15 × 10^6^ Tregs from WT and cKO mice. Mouse body weight was measured weekly (**D**), and frequency of IFN-γ–producing CD45.1^+^CD4^+^ T cells and mean fluorescence intensity (MFI) of IFN-γ in different organs in recipient mice was also measured (**E**). Data are representative of 3 independent experiments. Data are represented as means ± SD. Significance was measured by unpaired 2-tailed Student’s *t* test.

The preceding discoveries prompt the inquiry of whether Ku70 directly impacts the functionality of Tregs. To learn the effect of Ku70 on the function of Tregs, we generated mice with Treg-conditional knockout of Ku70 (*FOXP3^Cre–YFP^XRCC6^fl/fl^*, designated as cKO) by crossing *FOXP3^Cre–YFP^* (designated as WT) mice with *XRCC6^fl/fl^* mice (Supplemental Figure 2A). Validation of Treg-specific deletion of Ku70 was confirmed via Western blot analysis (Supplemental Figure 2, B and C). The development of T cells in the thymus of the cKO group was normal (Supplemental Figure 2D). However, we observed an increase in the proportion of central memory and effector memory CD4^+^ T cells in both the spleen and peripheral lymph nodes (pLNs) of cKO mice compared with WT mice. Additionally, the proportion of activated CD8^+^ T cells increased in the spleen of cKO mice compared with WT mice. The activated Treg proportion remained comparable (Figure 2A). As mice aged, progressive alterations in peripheral immune homeostasis were observed. At 6 weeks of age, the peripheral immune homeostasis of WT and cKO mice was comparable (Supplemental Figure 3, A–C). However, at the age of 14 weeks, a noteworthy reduction in the proportion of Tregs was detected in multiple tissues of cKO mice, concomitant with an increase in the proportion of IFN-γ^+^ Tconv in the lung, in contrast to their WT counterparts. This was accompanied by diminished expression levels of FOXP3 within the pulmonary tissue (Figure 2B and Supplemental Figure 3, D and E). By the age of 30 weeks, cKO mice demonstrated not only a sustained decrease in Treg proportions and an increase in lung IFN-γ^+^ Tconv cells, but also a significant elevation in the secretion of IFN-γ by CD8^+^ T cells in the spleen, pLNs, and lung (Figure 2C and Supplemental Figure 3, F and G). Although the absence of Ku70 in Tregs does not precipitate severe autoimmune inflammation in mice, the observed perturbations in immune homeostasis with advancing age across multiple tissues suggest that Ku70 deficiency compromises the suppressive capabilities of Tregs. Notably, the pulmonary tissue appears to be particularly susceptible to the functional alterations induced by the deficiency of Ku70 in Tregs.

Since Ku70 was associated with DNA damage, we also detected the DNA damage level of Tregs. In vitro treatment of natural Tregs (nTregs) sorted from WT and cKO mice with etoposide — a topoisomerase II inhibitor known to induce DNA DSBs — demonstrated that Tregs lacking Ku70 are more susceptible to the cytotoxic effects of high concentrations of DNA-damaging agents (Supplemental Figure 4, A and B). However, robust T cell receptor (TCR) activation in vitro did not markedly influence the DNA damage levels or the proliferative capacity of Ku70-deficient Tregs (Supplemental Figure 4, C and D). Furthermore, ex vivo analysis revealed that the DNA damage levels or the proliferative capacity in Tregs were comparable between WT and cKO mice (Supplemental Figure 4, E and F). Thus, we speculated that Ku70 directly regulates Treg function to affect immune homeostasis, especially in the lung.

We sorted naive T cells from WT and cKO mice to induce into iTregs in vitro and found the absence of Ku70 impedes the induction and differentiation efficiency of iTregs (Supplemental Figure 5A). Besides, Ku70 expression is upregulated during the generation of mouse iTregs (Supplemental Figure 5B). To further determine the impact of Ku70 deficiency on Treg function, we isolated CD45.2^+^ Tregs from WT and cKO mice and then transferred them along with naive CD45.1^+^CD4^+^ T cells into Rag1^–/–^ recipient mice to establish adoptive transfer colitis models. Our findings revealed that recipient mice engrafted with cKO Tregs, as opposed to those receiving WT Tregs, experienced slower weight gain and significant weight loss at 6 weeks (Figure 2D). At the 6 weeks following adoptive transfer, the proportion of cKO Tregs within the pLN and mesenteric lymph nodes (mLNs) of the recipient mice was notably reduced in comparison with that of WT Tregs (Supplemental Figure 5C). Moreover, recipient mice that received cKO Tregs demonstrated an elevated proportion of IFN-γ–producing cells in the pLN and a significantly heightened expression of IFN-γ within the transferred CD45.1^+^ cells in the colon and mLN (Figure 2E). These observations collectively indicate that mice with Tregs lacking Ku70 exhibit a disrupted immune homeostasis, particularly with advancing age, predominantly in the lung. This disruption is attributed to the compromised functionality of Tregs in the absence of Ku70.

### Ku70-deficient Tregs fail to suppress pulmonary antitumor immune responses.

**Figure 3:**
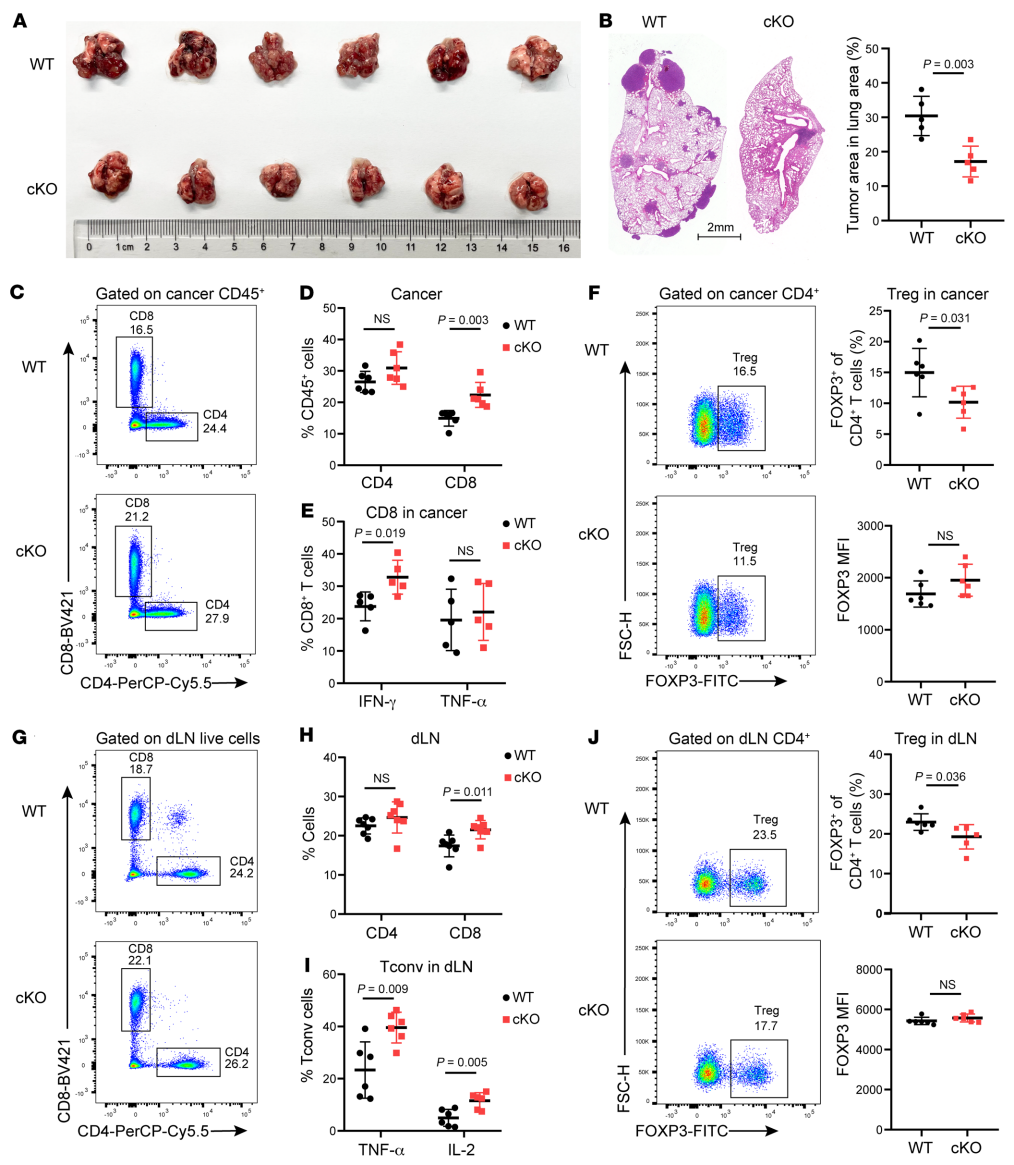
The absence of Ku70 in Tregs unleashes potent pulmonary antitumor immune responses. (**A**) Macroscopic evaluation of tumor size in lung from LLC-bearing WT and cKO mice (*n* = 6) at 21 days after tail-vein injection. (**B**) Representative lung histopathology images and percentage of lung area occupied by tumor lesions based on lung histopathological analysis (*n* = 5). (**C** and **D**) Representative histogram of CD4^+^ and CD8^+^ T cells (**C**) in lung cancer and summaries (**D**) for the frequencies (*n* = 6). (**E**) Frequency of IFN-γ– and TNF-α–producing CD8^+^ T cells in tumor of WT and cKO mice (*n* = 5). (**F**) Representative histogram of Treg proportion (left) in lung cancer and summaries (right) for the frequencies and Foxp3 MFI (*n* = 6). (**G** and **H**) Representative histogram of CD4^+^ and CD8^+^ T cells (**G**) in dLNs and summaries (**H**) for the frequencies (*n* = 6). (**I**) Frequency of TNF-α– and IL-2–producing Tconv cells in dLNs of WT and cKO mice (*n* = 6). (**J**) Representative histogram of Treg proportion (left) in dLNs and summaries (right) for the frequencies and Foxp3 MFI (*n* = 6). Data are represented as means ± SD. Significance was measured by unpaired 2-tailed Student’s *t* test.

To unravel the role of Ku70 in Treg function during tumor progression, we introduced Lewis lung carcinoma (LLC) into mice through tail-vein injection to establish a pulmonary tumor-colonizing model. In comparison with the WT group, cKO mice exhibited a notable reduction in lung tumor burdens (Figure 3A). Histopathological analysis of the lung tissue further revealed a diminished proportion of the lung area occupied by tumor lesions in cKO mice (Figure 3B). Additionally, our investigation unveiled that cKO mice had an augmented presence of intratumoral CD8^+^ T cells, accompanied by increased IFN-γ expression (Figure 3, C–E). Simultaneously, the percentage of tumor-infiltrated Tregs was lower in cKO mice than in WT mice (Figure 3F). These findings imply that the absence of Ku70 in Tregs compromises their ability to restrain the antitumor immune response. Moreover, the analysis of tumor-draining LNs (dLNs) indicated heightened levels of CD8^+^ T cells and reduced Tregs in cKO mice compared with WT mice. Furthermore, the Tconv in dLNs of cKO mice secreted higher levels of TNF-α and IL-2 (Figure 3, G–J).

**Figure 4:**
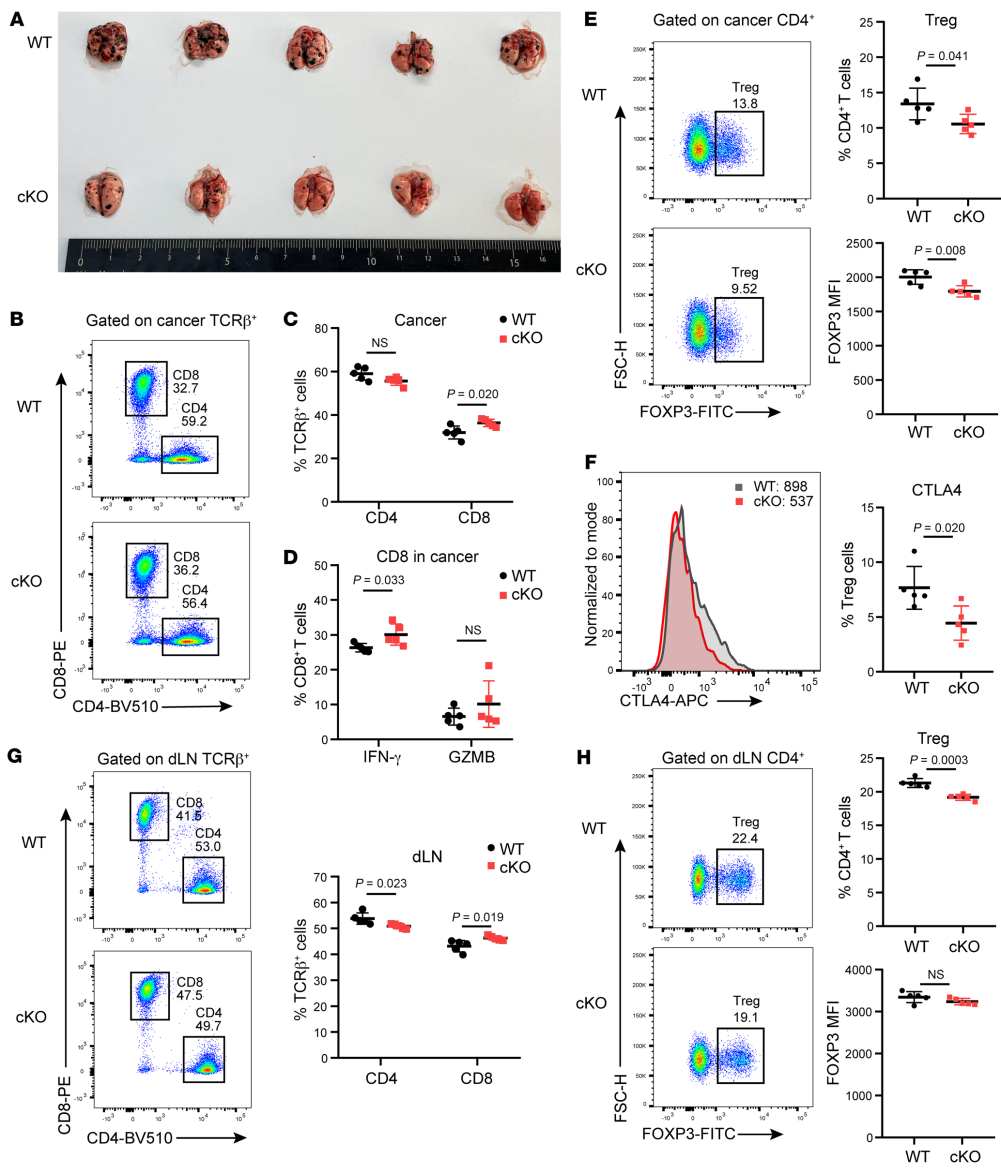
Tregs with Ku70 depletion trigger robust immune responses to restrain melanoma lung metastasis. (**A**) Macroscopic evaluation of tumor size in lung from B16F10-bearing WT and cKO mice (*n* = 5) at 21 days after tail-vein injection. (**B** and **C**) Representative histogram of CD4^+^ and CD8^+^ T cells (**B**) in lung cancer and summaries (**C**) for the frequencies (*n* = 5). (**D**) Frequency of IFN-γ– and GZMB-producing CD8^+^ T cells in tumor of WT and cKO mice (*n* = 5). (**E**) Representative histogram of Treg proportion (left) in lung cancer and summaries (right) for the frequencies and Foxp3 MFI (*n* = 5). (**F**) Representative histogram of CTLA4 expression (left) in lung cancer and proportion summaries (right) for CTLA4^+^ Tregs in tumor (*n* = 5). (**G**) Representative histogram of CD4^+^ and CD8^+^ T cells in tumor dLNs and summaries for the frequencies (*n* = 5). (**H**) Representative histogram of Treg proportion (left) in dLNs and summaries (right) for the frequencies and Foxp3 MFI (*n* = 5). Data are represented as means ± SD. Significance was measured by unpaired 2-tailed Student’s *t* test.

To verify the impact of Ku70-deficient Tregs on tumor, we also injected B16F10 cells into WT and cKO mice to develop a pulmonary metastasis model. The results coincided with the LLC model, and we similarly observed restrained tumor growth in Ku70-deficient mice (Figure 4A). Consistently, there was also an elevated proportion of intratumoral CD8^+^ T cells with increased IFN-γ expression in cKO mice compared with WT mice (Figure 4, B–D). Notably, the Treg percentages and FOXP3 expression in tumor were also lower in cKO mice than in WT mice (Figure 4E). Besides, the proliferation and the DNA damage marker γH2AX expression in T cells were comparable (Supplemental Figure 5, D and E). The in vitro treatment with tumor-conditioned medium (TCM) from LLC did not alter γH2AX expression in cKO mice (Supplemental Figure 5F). Interestingly, tumor-infiltrating Tregs in cKO mice exhibited reduced CTLA4 expression (Figure 4F), indicating compromised Treg function following Ku70 ablation. The dLNs from cKO mice in the B16F10 tumor mouse model also displayed increased CD8^+^ T cell infiltration and decreased Treg proportions (Figure 4, G and H). Collectively, these findings suggest that the deficiency of Ku70 in Tregs leads to effective pulmonary antitumor immune responses, resulting in slower tumor growth and increased infiltration of CD8^+^ T cells into the lung-colonizing tumors.

However, in the B16F10 subcutaneous tumor model, we did not observe a marked containment of tumor growth in cKO mice (Supplemental Figure 6, A–C). Additionally, the quantitative analysis of T cell populations and cytokine secretion within TME demonstrated no substantial differences between cKO and WT mice (Supplemental Figure 6, D and E). Previous literature has posited that the fitness of tumor-infiltrating Tregs is influenced by various factors within the TME (19, 20). Consistent with the elevated Ku70 expression observed in Tregs within LUAD, Tregs from mouse LLC lung-colonized tumors also exhibited higher Ku70 expression compared with Tconv cells (Supplemental Figure 7A). We explored the effect of TME factors on Ku70 expression in human iTregs. Our results indicated that TCM, particularly from LLC, substantially upregulated Ku70 expression in human iTregs (Supplemental Figure 7B). Among the various factors tested, glucose deprivation, but not TGF-β and CoCl_2_-induced hypoxia, was found to notably induce Ku70 expression in human iTregs (Supplemental Figure 7, C–E). These findings collectively suggest that the prognostic implications of Ku70 are intricately shaped by the distinct characteristics of the pulmonary TME, underscoring the complexity of Ku70’s role in modulating Treg behavior.

### Knockdown of Ku70 impairs the suppressive function of human iTregs.

**Figure 5:**
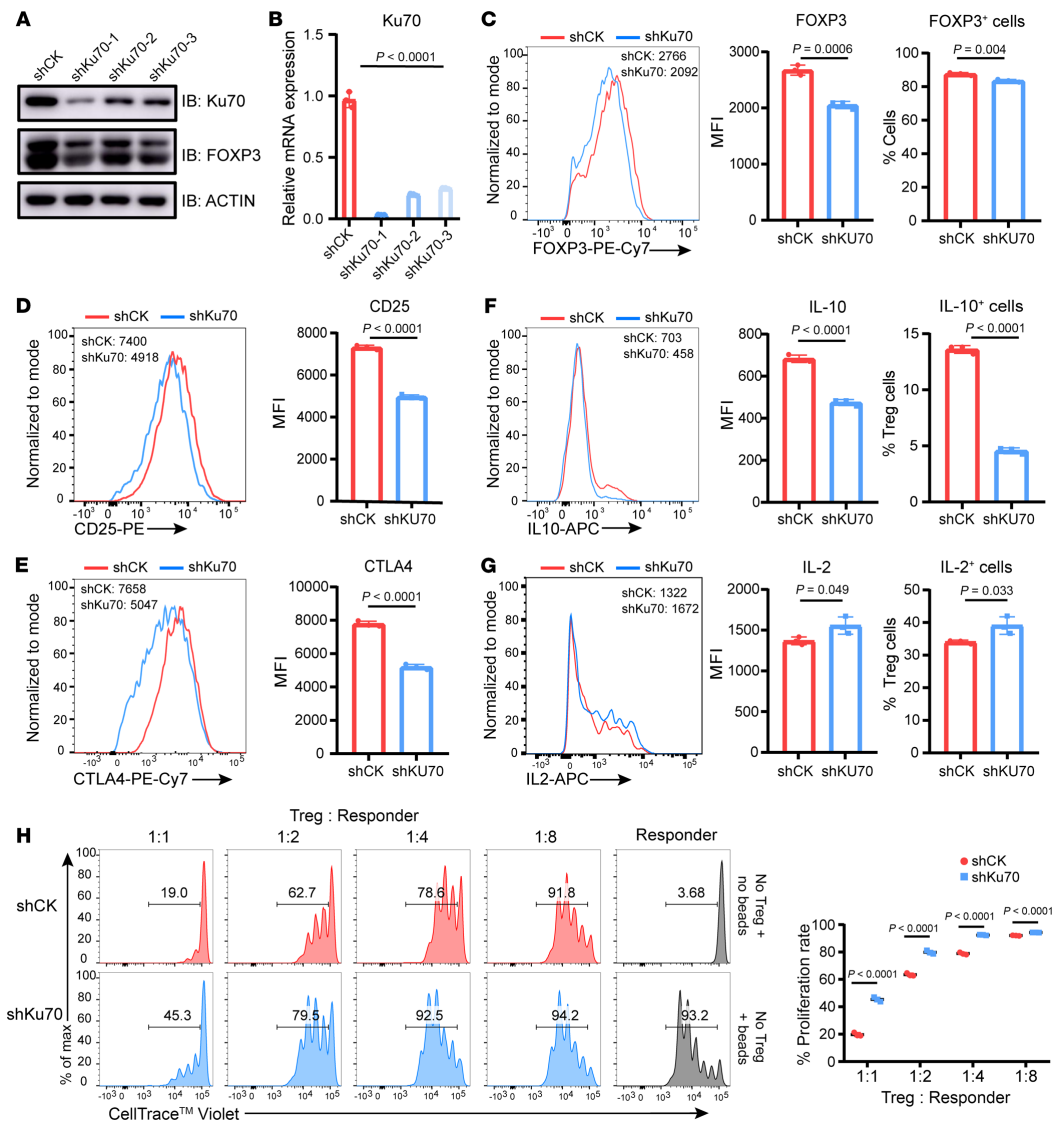
Human iTreg suppressive function is compromised upon knockdown of Ku70. (**A** and **B**) Ku70 knockdown assay was performed in human iTregs using shRNA-Ctrl (shCK) or shRNA-Ku70 (shKu70) lentiviruses, and the expression levels of Ku70 and FOXP3 were examined by Western blot (**A**) and qRT-PCR (**B**). (**C**–**E**) Representative histogram of protein abundance of FOXP3, CD25, and CTLA4 in control and shKu70 knockdown human iTregs and summaries for the MFI. (**F** and **G**) IL-10 and IL-2 production from human iTregs were assessed after knockdown of Ku70 by lentiviruses carrying Ku70 shRNA. (**H**) In vitro suppression assay was performed in human iTregs with or without Ku70 knockdown. Human iTreg and CD8^+^ T cells (responder) were cocultured for 84 hours at a ratio of 1:1, 1:2, 1:4, or 1:8 with anti-CD3/CD28 beads for activation. The responder groups include 2 control groups: one without anti-CD3/CD28 bead stimulation and one with anti-CD3/CD28 bead stimulation but without coculture with Tregs. The generations of proliferating responder were monitored by dye dilution of CellTrace Violet (CTV). Data are representative of 3 independent experiments. Data are represented as means ± SD. Significance was measured by unpaired 2-tailed Student’s *t* test.

To elucidate the impact of Ku70 on human Treg function, human iTregs differentiated from naive CD4^+^ T cells in vitro were infected with lentivirus carrying shRNA targeting Ku70 (shKu70) or scrambled control (shCK). shKu70-1 most effectively reduced the expression of Ku70 and was used for subsequent experiments (Figure 5, A and B). The data revealed that Ku70 knockdown resulted in a notable reduction in FOXP3 expression and the proportion of FOXP3^+^ cells (Figure 5C). Noteworthy alterations were also observed in the expression of suppressive function–associated proteins, including CD25, CTLA4, IL-10, and granzyme B (GZMB), all of which were downregulated following Ku70 knockdown (Figure 5, D–F, and Supplemental Figure 8A). Additionally, Tregs with Ku70 knockdown exhibited increased production of IL-2, a cytokine linked to the proliferation and differentiation of activated T cells (Figure 5G). To explore the role of Ku70 in Treg differentiation, we knocked down Ku70 in human naive CD4^+^ T cells, revealing a decline in the iTreg differentiation ratio (Supplemental Figure 8B). Ku70 expression is upregulated during the generation of human iTregs (Supplemental Figure 8C). Besides, Ku70 knockdown attenuated the expression of proliferation marker Ki67 (Supplemental Figure 8D). Consistent with these findings, in vitro suppressive assays demonstrated a marked impairment in the Treg capacity to suppress CD8^+^ T cell proliferation upon Ku70 knockdown (Figure 5H). Taken together, these observations strongly suggest that Ku70 plays an essential role in maintaining human Treg functions.

### Ku70 interacts with FOXP3.

**Figure 6:**
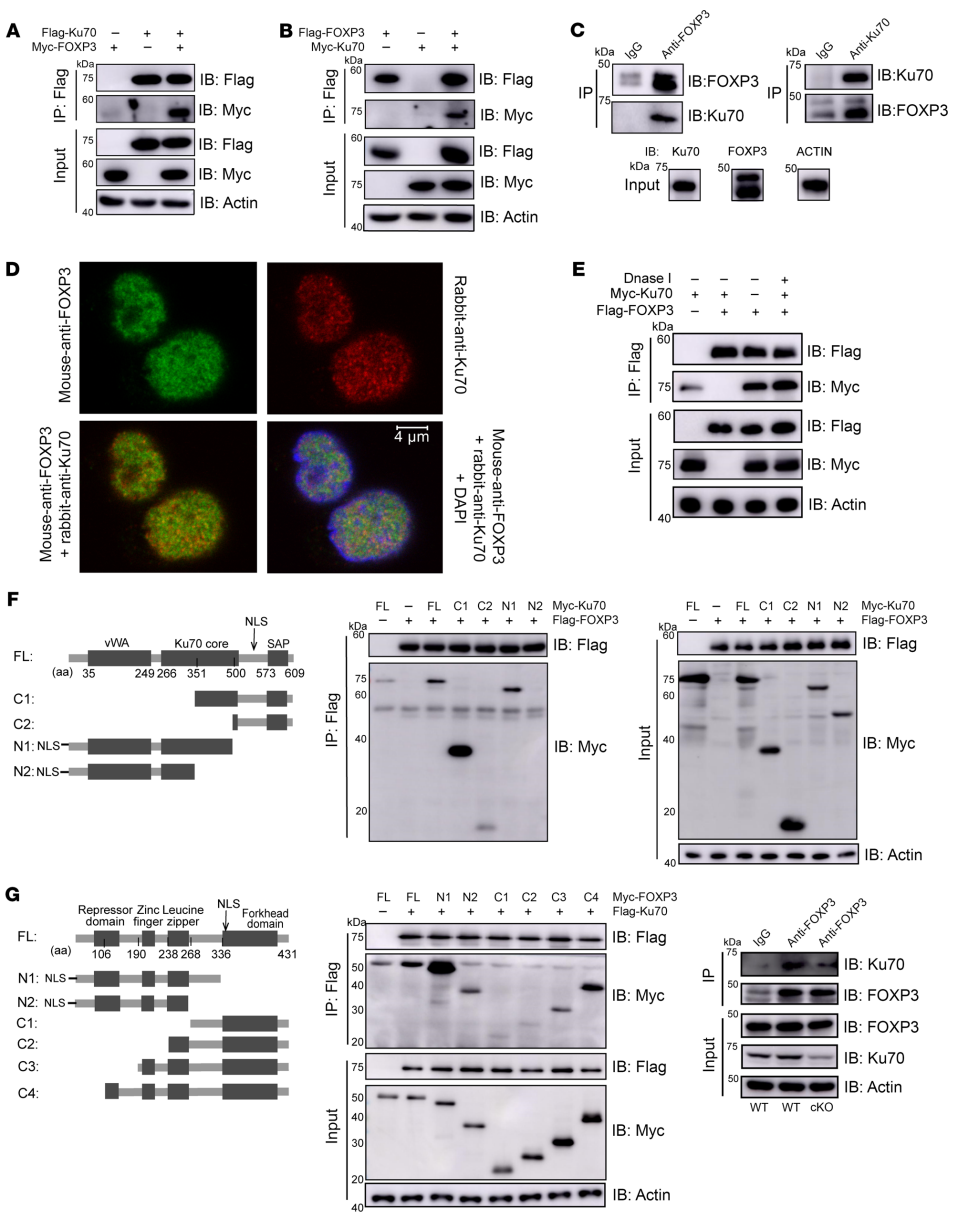
FOXP3 physically interacts with Ku70. (**A** and **B**) HEK293T cells were transfected with Flag-Ku70/Myc-FOXP3 (**A**) or Flag-FOXP3/Myc-Ku70 (**B**). Cell lysate was IP with anti-Flag antibody plus protein A/G beads. The results were analyzed by Western blotting using the indicated antibodies. (**C**) Endogenous IPs with anti-Ku70 or anti-FOXP3 antibody were performed in human iTregs induced in vitro. The results were detected by Western blotting with indicated antibodies. (**D**) IF analysis of Ku70 (red), FOXP3 (green), and DAPI (blue) in human iTregs after 24-hour activation by anti-CD3/CD28 beads. (**E**) HEK293T cells were transfected with Flag-FOXP3/Myc-Ku70. DNase I (10 U) were added to HEK293T lysates, which were subjected to IP using anti-FLAG antibody followed by Western blotting. (**F**) Schematic representations of the plasmids encoding full-length (WT) and truncated mutants of Ku70 (left). Flag-tagged FOXP3 was cotransfected into HEK293T cells with the indicated Myc-tagged Ku70 constructs and IP with anti-Flag antibody followed by Western blotting. vWA, von Willebrand A domain; NLS, nuclear localization signal; SAP, SAF-A/B, acinus and PIAS domain. Ku70 mutant constructs include Ku70-C1 (amino acids [aa] 351–609), Ku70-C2 (aa 500–609), Ku70-N1 (aa 1–500), and Ku70-N2 (aa 1–351), with each fused to a Myc tag. (**G**) Schematic diagram of FOXP3 and the deletion constructs (left). Flag-tagged Ku70 was cotransfected into HEK293T cells with the indicated Myc-tagged FOXP3 constructs and IP with anti-Flag antibody followed by Western blotting. (**H**) Naive CD4^+^ T cells were sorted from spleen of WT or cKO mice (8 weeks old), then were induced into iTregs in vitro. Endogenous IPs with anti-Foxp3 or IgG antibody were performed after 5 days of induced differentiation. The results were analyzed by Western blotting using the indicated antibodies.

Previous insights suggested that acetylated FOXL2, a member of the forkhead family, could restrict DSB repair by binding to Ku70 (21). In order to deeply investigate the regulatory role of Ku70 in Treg function, we explored its potential interaction with FOXP3. Co-immunoprecipitation (Co-IP) assays confirmed the interaction between Ku70 and FOXP3 in HEK293T cells, which were cotransfected with FOXP3 and Ku70 overexpression plasmids (Figure 6, A and B). The endogenous interaction of Ku70 with FOXP3 was also observed in human iTregs (Figure 6C). Immunofluorescence (IF) analysis revealed colocalization of Ku70 with FOXP3 in the nucleus of iTregs following 24 hours of TCR stimulation (Figure 6D). Notably, we demonstrated that Ku70-FOXP3 interaction was independent of DNA, as evident by adding DNase I in the cell lysate during the Co-IP assay (Figure 6E). Consequently, these results indicated that Ku70 is a part of the FOXP3 complex in human Tregs.

To pinpoint the FOXP3-binding domain of Ku70, we generated 4 Myc-tagged Ku70 deletion mutants. Co-IP experiments revealed that Ku70-C2 (500-609aa) and Ku70-N2 (1-351aa) lost the ability to bind FOXP3 (Figure 6F), indicating that FOXP3 binds to a specific region within the Ku70 core (351–500 aa). On the other hand, when we generated 6 truncated FOXP3 constructs with distinct domain depletions, only FOXP3-C1 (268–431 aa) and FOXP3-C2 (238–431 aa) truncations showed an inability to bind Ku70, while other truncated Myc-FOXP3 mutants retained their Ku70-binding ability (Figure 6G). This highlighted the critical role of the region (190–238 aa) containing the zinc finger for the interaction between FOXP3 and Ku70. Furthermore, we also uncovered an endogenous interaction between the Ku70 and FOXP3 in mouse iTregs (Figure 6H). These findings emphasized that the DNA repair protein Ku70, through its Ku70 core region, binds to the zinc finger of the transcription factor FOXP3, independently of DNA presence and across species.

### Ku70 supports the activity of FOXP3 transcription.

**Figure 7:**
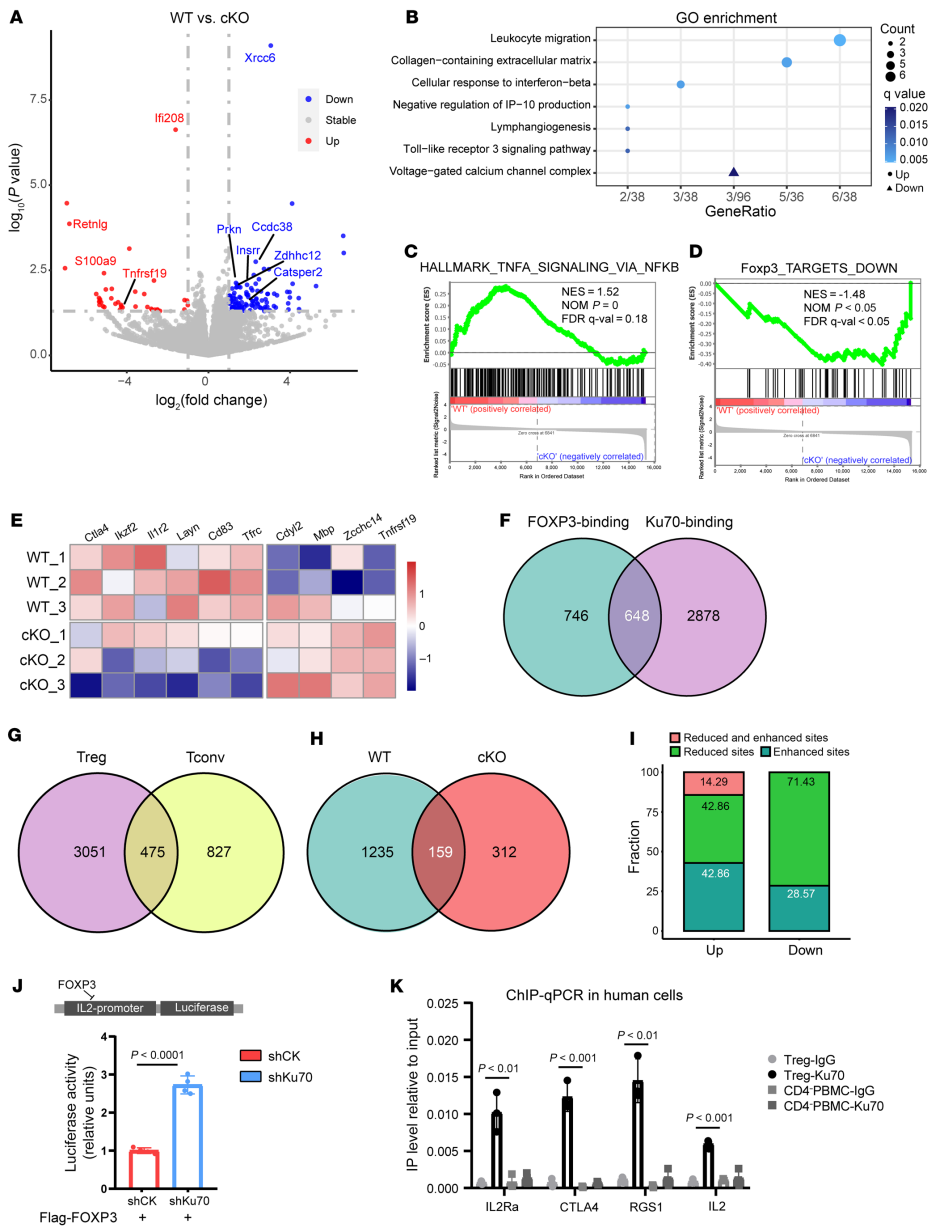
Ku70 enhances the functionality of FOXP3 transcription. (**A**) Volcano plot generated from RNA-Seq differential expression analysis using tumor-infiltrating Tregs. Tregs were isolated from LLC tumor-bearing WT or cKO mice at 21 days after tumor injection. Compared with the WT group, blue represents decrease in the cKO group, and red represents increase. The horizontal line denotes the significance threshold (*P* < 0.05) for DEGs. The vertical line denotes the log_2_(foldchange) threshold of 1. (**B**) Gene Ontology analysis of enriched pathways of upregulated genes (circle) or downregulated genes (triangle) in RNA-Seq. (**C** and **D**) GSEA enrichment plots of the indicated signatures in tumor-infiltrating WT and Ku70-deficient Tregs. (**E**) Heatmap showing expression of selected FOXP3-associated genes. (**F**) Venn diagram of FOXP3 ChIP-Seq peaks and Ku70 ChIP-Seq peaks in Tregs from *FOXP3*^Cre^ mice. (**G**) Venn diagram of Ku70 ChIP-Seq peaks in Tregs and Tconv cells from Foxp3Cre mice. (**H**) Venn diagram of FOXP3 ChIP-Seq peaks in Tregs from *FOXP3*^Cre^ and *FOXP3^Cre^XRCC6^fl/fl^* mice. (**I**) The bar graph illustrates the changes in FOXP3 binding for the differentially expressed genes that harbor altered FOXP3 binding sites. (**J**) HEK293T cells were cotransfected with either IL-2 promoter luciferase reporter and FOXP3-overexpressing plasmid, control vector, or shKu70 vector. Data were normalized to Renilla luciferase activity; the average induction in control group was set at 100%. (**K**) Anti-Ku70 or control rabbit Ig was used to precipitate crosslinked protein–DNA complexes from human iTreg lysates of CD4-negative (CD4^–^) PBMC lysates. The crosslinking of the immunoprecipitated material was removed and protease treated, and the DNA was purified. Ku70 IP versus the IgG fold enrichment ratio was determined from duplicate ChIP assay evaluated in duplicate by qRT-PCR. Data were normalized to input chromatin (normalized for nonspecific chromatin IP). *P* value was calculated between iTreg-Ku70 and CD4^–^ PBMCs. Data are represented as the mean ± SD. Significance was measured by unpaired 2-tailed Student’s *t* test.

To gain deeper insights into the impact of Ku70 knockout on Treg function, we performed RNA-Seq analysis of tumor-infiltrating Tregs sorting from lung-colonizing tumors. In comparing the cKO group to the WT group, we identified 39 upregulated genes and 110 downregulated genes in tumor-infiltrating Tregs (Figure 7A and Supplemental Table 3). Among the upregulated genes, we identified those associated with proinflammation and lymphocyte recruitment, such as interferon-activated gene 208 (*Ifi208*), resistin-like γ (*Retnlg*), and S100 calcium-binding protein a9 (*S100a9*). Conversely, downregulated genes were linked to diverse posttranslational modifications, including E3 ubiquitin-protein ligase *Prkn* and palmitoyltransferase *Zdhhc12*. Additionally, *Ccdc38*, a gene associated with lung function (22), exhibited decreased expression in the cKO group. Gene ontology analysis of the upregulated genes in the cKO group revealed enrichment in terms related to leukocyte migration, collagen-containing extracellular matrix, and cellular response to IFN-β, while the downregulated genes were enriched in the voltage-gated calcium channel complex (Figure 7B). Gene set enrichment analysis (GSEA) further highlighted the enrichment of TNFA_SIGNALING_VIA_NFKB term in the WT group compared with the cKO group (Figure 7C). Thus, these findings suggested that Ku70-absent Tregs may have an impaired ability to infiltrate tumors and activate pathways in response to inflammatory factors.

Notably, we observed an elevation in the expression of *Tnfrsf19*, a gene suppressed by Foxp3, in the cKO group (Figure 7A). Moreover, based on previous literature (23), a gene set of 48 genes bound and downregulated by FOXP3, termed FOXP3_TARGETS_DOWN, was enriched in the cKO group (Figure 7D). The heatmap shows that genes upregulated in tumor-infiltrating Tregs were reduced in cKO mice, while genes downregulated by FOXP3 exhibited increased expression in the cKO group (Figure 7E). To validate whether Ku70 directly regulates the transcriptional function of FOXP3, we conducted chromatin immunoprecipitation sequencing (ChIP-Seq) to investigate the genome-wide occupancy of FOXP3 and Ku70 in Tregs. Our analysis revealed that 46.48% of the FOXP3 peaks overlapped with Ku70 peaks in Tregs, and their genomic distribution was largely similar (Figure 7F and Supplemental Figure 9A). Compared with Tconv cells, 86.53% of Ku70-binding sites are unique to Tregs (Figure 7G). Further, we evaluated the influence of Ku70 deficiency on the binding patterns of FOXP3. The assay revealed that 88.59% of FOXP3 peaks in WT Tregs were markedly reduced in intensity in Ku70-deficient Tregs, with 34% of these peaks corresponding to gene loci also occupied by Ku70 (Figure 7H and Supplemental Figure 9B). Additionally, we integrated RNA-Seq results and assessed the genes possessing the altered FOXP3-binding sites among differential genes; 18% up genes and 6% of down genes exhibited altered FOXP3 binding. Among the downregulated genes, 71.43% of the genes exhibited decreased Foxp3 binding (Figure 7I). These results indicate that FOXP3 and Ku70 share a substantial number of target genes in Tregs, suggesting that Ku70 can directly influence the binding of FOXP3 to the genome.

Additionally, in human iTregs, the knockdown of Ku70 resulted in a reduced transcription level of signature molecules directly regulated by FOXP3, such as CD25 and CTLA4 (Supplemental Figure 9C). The double luciferase reporter gene experiment showed that Ku70 knockdown markedly disrupted the repression of FOXP3 on IL-2 promoter–mediated luciferase transcription (Figure 7J). Utilizing ChIP-qPCR, we analyzed the binding of Ku70 at FOXP3-occupied sites (24, 25). Compared with the CD4-negative PBMCs and Ig isotype, Ku70 in human iTregs is significantly bound to FOXP3-bound chromatin regions, such as IL-2Ra, CTLA4, RGS1, and IL-2 promoter regions (Figure 7K). These data indicate that the binding is not due to the potential of Ku70 binding dsDNA and is dependent on the presence of FOXP3. Consequently, we thought that the Ku70-forming complex with FOXP3 supports the activity of FOXP3 transcription, shedding light on the underlying cause for the weakened suppressive function of Tregs following Ku70 disruption.

## Discussion

The DNA repair protein Ku70 has been widely reported to affect the resistance of cancer cells to chemicals and radiotherapy. However, its multifaceted nonclassical roles have gradually come into focus. As major barriers to antitumor immunity, Tregs emerge as promising targets for tumor immunotherapy. In this study, we reveal that Ku70 supports Treg-suppressive function by binding to FOXP3 and regulating the transcriptional activity of FOXP3. The disruption of Ku70 in Tregs inhibits pulmonary tumor growth, which is accompanied by an increased infiltration of CD8^+^ T cells. This underscores the nonclassical function of Ku70 in the immune system, which indicates that targeting Ku70 can combine its function in immune regulation with its DNA repair capabilities to exert control over pulmonary tumor progression.

While Ku70 is well known for its role in DNA repair, accumulating evidence suggests its influence extends beyond this function to regulate various crucial cellular activities. Among these, Ku70 is implicated in antiapoptotic mechanisms and gene transcription. Notably, cytosolic Ku70 has been observed binding to the proapoptotic protein Bax, thereby dampening Bax-induced cell death (13). Additionally, Ku70 serves as a deubiquitinase, stabilizing the antiapoptotic Mcl-1 (26). In human macrophages, Ku70 and Ku80 interact with the NF-κB complex following lipopolysaccharide (LPS) treatment, facilitating LPS-induced NF-κB activation and proinflammatory cytokine production (27). Moreover, Ku70 demonstrates an ability to read the epigenomic signature of acute enhancers, forming the Ku70–HP1γ–Med26 complex to promote transcriptional activation (28). In breast cancer cells, Ku70 collaborates with AP2, being recruited to the proximal promoter of ERBB2 for transcription regulation (29). Our research delineates an NHEJ-independent role for Ku70 in supporting FOXP3 transcriptional activity, highlighting Ku70’s pivotal involvement in immune regulation. It is noteworthy that humans have a longer lifespan compared with mice and exhibit significantly higher levels of DNA repair proteins, including Ku70 (2, 30). In Ku70-knockdown human iTregs, we observed more pronounced phenotypic alterations relative to murine Tregs. In murine Tregs, in both ex vivo and in vitro TCR stimulation, the absence of Ku70 did not elicit detectable changes in proliferation or DNA damage; in contrast, a reduction in Ki67 expression surfaced in Ku70-knockdown human iTregs under in vitro TCR activation conditions. These observations suggest that Ku70 plays a more critical role in sustaining the functionality of human Tregs. The acquisition of additional clinical data is imperative to further elucidate the influence of Ku70 regulation on Tregs in human diseases. Moreover, our research has validated alterations in the characteristics of Tregs following Ku70 absence and the interaction between Ku70 and FOXP3, thereby highlighting the existence and importance of Ku70’s noncanonical functions. To enhance our comprehension of this nontraditional role, further investigation, potentially involving the exploration of small molecules capable of intervening in the Ku70-FOXP3 interaction, is warranted and holds marked potential for advancing therapeutic strategies.

The association between Ku70 and tumor prognosis is organ specific; generally, high expression of Ku70 in cancer cells correlates with adverse clinicopathological and enhanced chemotherapy resistance (2, 31–33). Research found that Ku70 binds to FOXO4, confining FOXO4 within the nucleus, which disrupts its tumor-suppressor ability and promotes cancer cell proliferation (34, 35). However, intriguingly, in head and neck cancer, higher Ku70 mRNA levels were found in chemotherapy responders compared with nonresponders (36). During colitis, increased cytosolic DNA induces the translocation of Ku70 to the cytosol, where it forms a complex with GTPase Ras and kinase Raf, activating the MEK/ERK pathway to inhibit intestinal tumor formation (37). In this study, we have elucidated that ablation of Ku70 in Tregs leads to altered lung immune homeostasis and enhanced pulmonary antitumor immune responses. Nonetheless, the absence of Ku70 in Tregs fails to inhibit the growth of the B16F10 subcutaneous tumor model. An analysis of TCGA data revealed that *XRCC6* overexpression was associated with an unfavorable prognosis in LUAD, while no such correlation was found in LUSC, COAD, and LIHC. Our research corroborated that the absence of Ku70 in Tregs diminishes their immunosuppressive properties. The findings from Co-IP and ChIP elucidated that Ku70 forms a complex with FOXP3 to regulate the transcription of FOXP3 target genes. This interaction is not tissue specific and represents a general mechanism that influences Treg functionality across various tissues. However, a heightened sensitivity to Ku70 regulation was observed in lung Tregs. The underlying factors of this increased sensitivity are currently not fully understood. Within TME, we found that TCM from LLC substantially upregulated Ku70 expression in human iTregs when compared with TCM from B16F10. Moreover, glucose deprivation, rather than TGF or hypoxia, was identified as a marked enhancer of Ku70 expression in human iTregs. These findings suggest that the metabolic heterogeneity within different tumor types may account for the divergent behaviors of Ku70-deficient Tregs in various malignancies. A profound comprehension of the distinct roles of Ku70 across various cancers will aid in better targeting Ku70 for tumor therapy.

Lung cancer remains the leading cause of global cancer mortalities, though immunotherapy brings encouraging therapy benefit. Increasing evidence underscores the pivotal role of antitumor immune activation in controlling tumor evolution. Targeting immunosuppressive Tregs, such as through the neutralization of CTLA-4, has been recognized as an effective strategy to enhance the antitumor immune response. However, existing research has demonstrated that in “cold” tumor models characterized by low lymphocyte infiltration, such as the TC-1 lung tumor model, radiotherapy enhances the expansion and activation of tTregs (Helios^+^), which possess an effector phenotype. And combining with CTLA4 blockade further augments the activation of these effector phenotype Tregs, ultimately failing to control tumor growth (38). These findings suggest that radiotherapy-induced DNA damage and the subsequent DNA repair pathways may exert a more intricate influence on the behavior and function of Tregs. Many molecules, acting as cofactors of FOXP3, play a crucial role in regulating the stability of Treg function, including NFAT (39), RUNX1 (40), and DBC1 (41). In our research, we found that the interaction of DNA damage repair protein Ku70 and FOXP3 influences the transcription of Treg-specific features, which shed light on the intricate mechanisms of FOXP3-dependent regulation. Additionally, we have previously reported that poly (ADP-ribose) polymerase 1 (PARP1), a key enzyme in initiating various forms of DNA repair, enhances FOXP3 poly(ADP-ribosyl)ation, thereby suppressing the suppressive activity of Tregs (42). These findings suggest that DNA repair pathways may harbor novel roles in immune regulation, opening up new possibilities for intervening in DNA repair proteins to boost the antitumor immune response and dampen cancer cells themselves as a potential approach for tumor treatment.

The impact of the interaction may be reciprocal. While our findings focused on Ku70 binding to FOXP3 to enhance FOXP3’s transcriptional activity, we did not delve into comprehensive research on whether FOXP3 influences the DNA repair activity of Ku70. Regarding the role of FOXP3 in DNA repair, in cancer cells, the absence of FOXP3 enhances the homologous recombination (HR) pathway by activating BRCA1 expression (43). In cancer cells subjected to DNA damage stimuli, FOXP3 expression is induced in a p53-dependent manner (44). However, as a critical transcription factor of Tregs, the role of FOXP3 in Treg DNA repair is unclear, with conflicting studies on Treg resistance to DNA-damaging drugs. Some papers suggest that both human and murine Tregs are more resistant to DNA damage agents (45, 46), while others show a decrease in Treg proportion in a mouse tumor model after chemotherapy drug treatment (47). Further research, particularly involving clinical data, is essential for elucidating the role of FOXP3 in NHEJ and assessing the resistance of various T cell clusters to DNA-damaging agents.

Furthermore, despite observing age-related alterations in immune homeostasis across multiple tissues in cKO mice, severe inflammatory responses were not detected in these mice. Our research primarily focuses on the functional changes of Ku70-deletion Tregs in the TME, with a current lack of comprehensive insight into the implications of Ku70 deficiency in Tregs under conditions of autoimmune inflammation. We have established adoptive transfer colitis models and discerned that Tregs deficient in Ku70 markedly diminish their capacity to regulate the progression of colitis. However, it is not yet clear whether the accumulation of cytosolic DNA, potentially resulting from autoimmune inflammation, triggers changes in the cellular localization of Ku70, thereby influencing the immunosuppressive function of Tregs in a manner that is independent of FOXP3.

Existing data have already proposed an essential role of Ku70 in chemoresistance. For example, Ku70 knockdown significantly facilitated human cancer cell sensitivity to radiation and the topoisomerase II inhibitor (2). Our results suggest that Ku70 deficiency damages the suppressive ability of Tregs and promotes pulmonary antitumor immunity. In conclusion, these findings highlight that the DNA repair pathway may regulate tumor immune response through nonclassical roles to affect the tumorigenesis and progression of lung cancer.

## Methods

### Sex as a biological variable.

Our study examined male and female animals, and similar findings are reported for both sexes.

### Samples of patients.

A total of 16 treatment-naive patients who were undergoing surgery, pathologically diagnosed with primary LUAD at Shanghai Chest Hospital, were enrolled in this study. Paired, fresh tumor and peritumor tissue samples (at least 2 cm from matched tumor tissues) were collected. We used the eighth American Joint Committee on Cancer TNM Staging (AJCC, 8th edition) (48) to classify the tumor. We excluded patients who fit the following criteria: (a) patients with a history of concurrent malignant disease or other previous primary cancers; (b) patients with a treatment history of preoperative induction therapy; (c) patients with nonclinical N0 disease, which is defined as preoperative CT scans showing hilar or mediastinal nodes with a shortest diameter of less than 1.0 cm; and (d) patients with nodules containing a ground-glass opacity (GGO) component in CT imaging. General clinical and pathological information are shown in Supplemental Tables 1 and 2.

### Mice.

All mouse lines in this paper were maintained on a C57BL/6J background. *Foxp3*^Yfp–Cre^ mice were purchased from the Jackson Laboratory (no. 016959). *Xrcc6*^fl/fl^ mice were purchased from Saiye Biotechnology Co. Ltd. All mice received standard care in a specific pathogen–free environment (84–86°F, 55% humidity, 12-hour light/12-hour dark cycle, 22°C), with free access to food and water. To exclude cage effects, heterozygotes were bred to generate Treg-specific knockout mice (*Xrcc6*^fl/fl^*Foxp3*^Yfp–Cre^, cKO) and *Foxp3*^Yfp–Cre^ (control, WT) mice. The offspring mice aged 6–30 weeks were selected for this study, with the specific age of each experimental group noted in the figure legends. DNA was extracted from murine tail biopsy, then was used to perform PCR for genotyping. The genotyping primers are provided in Supplemental Table 4. The ages and numbers of mice per experimental group are indicated in the figure legends.

### Plasmids and antibodies.

Pip-Flag/Myc–tagged FOXP3 and FOXP3 truncations were previously constructed in our lab (49). Flag/Myc–tagged Ku70 and Ku70 truncations were constructed into pip-plasmids. For knockdown assay, targeted Ku70 shRNAs or scrambled shRNA was cloned into pLKO.1-GFP lentiviral vector. shKu70 target sequence and primers for plasmid construction are provided in Supplemental Table 4. Antibodies used for IP, IF, immunoblotting (IB), and flow cytometry are provided in Supplemental Table 5.

### Immunohistochemical and H&E staining.

Partial samples from patients were fixed by formalin, then embedded by paraffin for IHC to detect Ku70 levels in tumors and paired peritumor tissues. The paraffin section was used for IHC staining for Ku70 (Proteintech: 10723-1-AP). Quantification of IHC staining was evaluated by IHC profiler plugin in ImageJ software (NIH). For the mouse tumor model, the right middle lobes of lung tissues from the tumor model were embedded in paraffin and processed for H&E staining to detect tumor lesions.

### Isolation of murine T cell subsets and murine iTreg induction.

Murine lymphocytes were isolated from the spleen and pLNs. CD4^+^ cells were purified by murine CD4 MicroBeads (Miltenyi Biotec, catalog 130-049-201). Murine naive CD4^+^ T cells (CD4^+^CD25^–^YFP^–^CD62L^+^), Tregs (CD4^+^CD25^+^YFP^+^), and Tconv cells (CD4^+^CD25^–^YFP^–^) were sorted by BD FACS Aria II Cell Sorter. Isolated mouse T cells were cultured in RPMI 1640 medium (Gibco, Thermo Fisher Scientific, C22400500) supplemented with 10% FBS (Gibco, Thermo Fisher Scientific, 10100147), 1% sodium pyruvate (Gibco, Thermo Fisher Scientific, 11360070), 1% MEM Non-Essential Amino Acids Solution (Gibco, Thermo Fisher Scientific, 11140050), and 1% penicillin–streptomycin (Gibco, Thermo Fisher Scientific, 15140122). For murine iTreg induction, naive CD4^+^ T cells were cultured with 5 μg/mL plate-coated antibody anti-CD3 (Bio X Cell, BE0001-1) and 3 μg/mL soluble antibody against CD28 (Bio X Cell, BE0015-1) in the presence of 100 U/mL IL-2 (R&D Biotech, 402-ML) and 5 ng/mL TGF-β (R&D Biotech, 7666-MB-005/CF) for 3 days.

### Cell culture and transfection.

HEK293T, LLC, and B16F10 were purchased from ATCC and were grown in DMEM (Gibco, Thermo Fisher Scientific, C11995500) supplemented with 10% FBS. Cells were cultured in cell incubator at 37°C with 5% CO_2_. HEK293T cells were transiently transfected with plasmids using polyethylenimine (PEI) (Polysciences, 23966-2) protocols. After about 40 hours of transient transfection, the cells were collected for experiments. For lentiviral packaging, the mixtures containing PEI/pLKO.1/dR8.9/VSVG were transfecting into HEK293T cells. The supernatant was collected at 48 and 72 hours after transfection.

### Isolation of human naive CD4^+^ T cells, Tregs, and CD8^+^ cells.

Human PBMCs were isolated from peripheral blood of healthy donors from Shanghai Blood Center. Human total CD4^+^ and CD8^+^ T cells were purified by human CD4 MicroBeads (Miltenyi Biotec, 130-045-101) and human CD8 MicroBeads (Miltenyi Biotec, 130-045-201), respectively. Human naive CD4^+^ T cells (CD4^+^CD25^lo^CD127^hi^CD45RA^+^), Tregs (CD4^+^CD25^hi^CD127^lo^), and Teff cells (CD4^+^CD25^lo^CD127^hi^) were sorted using the BD FACS Aria II Cell Sorter.

### Human iTreg induction and lentivirus infection.

Human naive CD4^+^ T cells were cultured in X-VIVO medium (Lonza, 04-418Q) containing 10% FBS, 1% GlutaMAX (Gibco, Thermo Fisher Scientific, 35050061), 1% sodium pyruvat, 1% MEM nonessential amino acids solution, and 1% penicillin–streptomycin, in the presence of Dynabeads Human T-activator CD3/CD28 (Gibco, Thermo Fisher Scientific, 11132D), treated by 100 U/mL human IL-2 (PeproTech, 200-02) and 5 ng/mL human TGF-β (R&D Systems, 7754-BH) to induce human iTregs in vitro for 7 days. For lentivirus infection, after about 5 days inducing, the lentiviral supernatants concentrated by Polyethylene Glycol 8000 (MilliporeSigma, P2139) replaced the medium of Tregs supplemented with 8 μg/mL Polybrene (MilliporeSigma, TR-1003-G). Then the mixtures were spun in a benchtop centrifuge at 975*g* or 120 minutes at 32°C. The mixtures were maintained for 12 hours in cell incubators. Lentivirus-transduced GFP^+^ Tregs were sorted using the BD FACS Aria II cell sorter.

### Flow cytometry and cytokines stimulation analysis.

Cells isolated from tissues were washed in FACS buffer (PBS containing 2% FBS and 2 mM EDTA) twice. For analysis of surface markers, cells were stained in FACS buffer with indicated antibodies for 30 minutes. FOXP3 staining was performed according to the manufacturer’s instructions (Foxp3/Transcription Factor Staining Buffer Set, eBioscience, 00-5523-00). For cytokine analysis, cells were prestimulated in culture medium with phorbol 12-myristate 13-acetate (50 ng/mL, MilliporeSigma, P1585-1MG), ionomycin (1 μM, MilliporeSigma, I3909-1ML), and Golgi Stop (BD Pharmingen: 554724) for 5 hours.

### Tumor model.

To establish lung metastasis model, LLC cells (1 million per mouse) or B16F10 cells (0.35 million per mouse) were tail intravenously injected into 8- to 10-week-old WT and cKO mice. Tumors were excised at 21 days and were digested for 30 minutes at 37°C with Collagenase IV (1 mg/mL, MilliporeSigma, C5138-1G), and DNaseI (0.15 mg/mL, MilliporeSigma, DN25-100mg) for flow cytometric analysis of the tumor microenvironment. For the subcutaneous tumor model, B16F10 cells (0.3 million per mouse) were injected into the flanks of 8- to 10-week-old WT and cKO mice. All mice were killed by cervical dislocation on day 15 after injection. Subcutaneous tumors were surgically excised, weighed, and photographed.

### Adoptive transfer colitis model.

CD45.2^+^ Tregs were sorted from the spleens of 8-week-old WT and cKO mice. Recipient Rag1^–/–^ mice were adoptively transferred intravenously with 0.45 × 10^6^ naive CD45.1^+^CD4^+^ T cells alone or coinjected with 0.15 × 10^6^ Tregs. Mice were monitored for weight and other clinical parameters as previously reported. The genotype of the recipient mice was analyzed 6 weeks after injection.

### In vitro suppression assay.

Sorted human CD8^+^ T cells were labeled with 2.5 μM CellTrace Violet (Invitrogen, C34557) for 15 minutes at 37°C. Human iTregs and labeled CD8^+^ T cells (responder) were cocultured at a ratio of 1:1, 1:2, 1:4, or 1:8 with anti-CD3/CD28 beads for activation. The responder groups included 2 control groups: one without anti-CD3/CD28 bead stimulation and one with anti-CD3/CD28 bead stimulation but without coculture with Tregs. Cells were collected after 84 hours of coculture for examining CD8^+^ T cell proliferation by BD LSRFortessa X-20.

### In vitro Treg treatment experiment.

nTregs were sorted from the spleens of 8-week-old WT and cKO mice and treated with Dynabeads Mouse T-activator CD3/CD28 (Gibco, Thermo Fisher Scientific, 11453D) or Etoposide (Selleck, S1225) for 24 or 48 hours in vitro. The expression of DNA damage marker γH2AX and proliferation marker Ki67 were detected by flow cytometry. The experiment on the effects of TCM stressors on Ku70 expression in human iTregs was conducted similarly to what was previously described (19). Human iTregs were washed and rested for 6 hours in X-Vivo medium. The culture supernatant DMEM medium from B16 and LLC cells, collected after 16 hours of incubation, was used as TCM. TCM or control DMEM medium was then mixed 50:50 with X-vivo medium and incubated on human iTregs for 24 hours. Human iTregs were incubated in X-Vivo containing IL-2 with or without the addition of TGF-β (16 ng/mL) or CoCl_2_ (0, 100, 200 μM) for 24 hours. The expression of Ku70 was detected by quantitative reverse-transcription PCR (RT-qPCR).

### IP and Western blot.

Cells were harvested, washed with PBS, and lysed on ice for 45 minutes in RIPA lysis buffer with proteinase inhibitors and phosphatase inhibitors. Cell lysates were centrifuged at 12,000 rcf, and then 1 μg of the antibodies was added into the supernatants and incubated at 4°C for 3 hours, followed by incubating with 12 μL of protein A/G Plus-agarose (Santa Cruz Biotechnology Inc., sc-2003) at 4°C for 1 hour. After washing, the precipitates were then subjected to SDS-PAGE and immunoblot analysis.

### Immunofluorescent staining.

Human iTregs were seeded in poly-l-lysine–coated glass coverslips and fixed by 4% paraformaldehyde for 15 minutes. Samples were permeabilized for 10 minutes with 0.2% Triton X-100 in PBS and blocked with 1% bovine serum albumin for 1 hour. Primary antibodies against Ku70 and FOXP3 were incubated at 4°C for 12 hours. After washing, samples were then incubated with secondary antibodies and DAPI for 1 hour at room temperature. A Leica SP8 confocal laser scanning microscope was used to observe the immunofluorescent intensity.

### RT-qPCR and bulk RNA-Seq analysis.

Total RNA was extracted using TRIzol reagent (Ambion, 15596018). Reverse transcription of RNA to cDNA was performed using the Reverse Transcriptase Kit (Vazyme, R323-01). RT-qPCR was run using SYBR qPCR Master Mix (Vazyme, Q711-02) on a ViiA7 PCR system. β-Actin was used as the reference gene. Primers used for RT-qPCR are listed in Supplemental Table 4. For RNA-Seq of tumor-infiltrating Tregs, the library construction and sequencing were conducted by Huada Corporation using the BGI-Seq500 sequencing platform. Paired-end sequence reads of 150 bp length were generated, and the clean reads were aligned to GRCm38. Gene counts were calculated and then imported into the R (version 4.1.2) for analyzing the differential gene expression by DESeq2 package (1.34.0). Significantly differentially expressed genes were defined by |log_2_FoldChange| ≥1 and *P* < 0.05 (Supplemental Table 3). Heatmap plots were generated with the R package pheatmap.

### ChIP assay for sequencing.

CD4^+^CD25^–^YFP^–^ Tconv and CD4^+^CD25^+^YFP^+^ Tregs were sorted from Foxp3^Yfp–cre^ mice for Ku70 ChIP-Seq, and Tregs were sorted from WT and cKO mice for FOXP3 ChIP-Seq. The ChIP-Seq experiments were performed with 2 biological replicates. Cells were crosslinked and the chromatin sonicated into fragments. Chromatin digestion was performed as described in the SimpleChIP Plus Enzymatic Chromatin IP Kit (Cell Signaling Technology, 9005) manual. The sheared chromatin was immunoprecipitated with antibodies against FOXP3, Ku70, or mouse control IgG overnight at 4°C, followed by precipitation with ChIP-Grade Protein G Magnetic Beads (Cell Signaling Technology, 9006S). The pulled-down DNA fragments and input samples were subjected to sequencing. Sequencing was conducted on the BGI DNBSEQ-T7 platform with 150 bp paired-end reads.

### Analysis for ChIP-Seq.

The quality of raw sequence reads was controlled using FastQC, and low quality reads (Phred score below 20) were discarded. The quality of raw sequence reads was controlled using FastQC, and low quality reads (Phred score below 20) were discarded. Raw sequences were trimmed using trim_galore with the default setting. Sequencing reads were mapped to the mouse genome mm10 using Bowtie2 with default setting. Duplicated sequences were removed by picard from GATK toolkit. The coverage and quality of the sequencing data are summarized in Supplemental Table 6. Common ChIP-Seq peaks in the given conditions were identified by merging 2 replicates for each condition, and were defined using MACS2 (50) callpeak method with the following command: macs2 callpeak -t ${name}_rep1.dedup.bam ${name}_rep2.dedup.bam -c input ${name}.dedup.bam -g mm -n ${name} -B -p 0.05, –nomodel -extsize 140. The Bdg2bw tool was used for the conversion of bedgraph to bigwig for visualization. Specific peaks between given conditions were identified by the MACS2 bdgdiff method as follows: macs2 bdgdiff --t1 ${condition1}_treat_pileup.bdg --c1 ${condition1}_control_lambda.bdg --t2 ${condition2}_treat_pileup.bdg --c2 ${condition2}_control_lambda.bdg -g 60 -l 140 --o-prefix diff. Overlaps between Chip-Seq peak and differentially expressed genes in RNA-Seq data were calculated using annotatePeak in R package ChIPseeker (51) with tssRegion being 3kb upstream or downstream. Venn plots were also generated with this condition. Integrated genome viewer (IGV) was used for the visualization of peak or region data.

### ChIP assay for qPCR analysis.

Human iTregs were induced and CD4^–^ PBMCs were isolated from healthy donors as previously described. ChIP assay was performed as previously described. The sheared chromatin was immunoprecipitated with antibodies against Ku70 or rabbit control IgG overnight at 4°C. The pulled-down DNA fragments were subjected to qPCR analysis with the primers listed in Supplemental Table 4.

### Luciferase-based transcription activity and repression assays.

The IL-2 luciferase reporter plasmid was cotransfected with a Renilla luciferase-encoding plasmid and a FOXP3-overexpressing plasmid into HEK293T cells, which were transfected with control vector or shKu70 vector. The cells were lysed and analyzed using a luciferase assay, and the results were normalized to Renilla luciferase activity according to the manufacturer’s protocol (Beyotime, RG027).

### Statistics.

Unless stated otherwise, data are represented as mean ± SD and *P* values were calculated with unpaired 2-tailed Student’s *t* test in GraphPad Prism, version 6.0. For RNA-Seq data, *P* values were adjusted using the Benjamini–Hochberg method.

### Study approval.

This study was approved by the Ethics Committee of Shanghai Chest Hospital (KS22026) and complied with all relevant ethics regulations; informed consent was obtained from each patient. Human PBMCs were isolated from peripheral blood of healthy donors from Shanghai Blood Center, which was approved by the Shanghai Blood Center Ethics Committee. All animal experiments followed the protocols approved by the Institutional Animal Care and Use Committee at the Institute of Shanghai Immunology, School of Medicine, Shanghai Jiao Tong University, under protocol number A-2022-031.

### Data availability.

The bulk RNA-Seq and ChIP-Seq data generated in this paper are available through the NCBI’s Gene Expression Omnibus database (GEO GSE247031 and GSE278950). Other data analyzed during this study are available from the GEO; accession numbers are listed in Figure 1. Values for all data points in graphs are reported in the Supporting Data Values file.

## Author contributions

QH led the project design, conducted experiments, analyzed the data, and wrote the manuscript. NT was primarily responsible for conducting experiments and interpreting the data. JZ contributed to the collection of clinical samples. SS contributed to data analysis. HC, XL, and WZ integrated and analyzed the data. YY and YD provided technical support. XD, RL, DL, and SMD provided technical support and revised the manuscript. CW, ZC, QZ, and BL supervised the project and provided technical and material support. All authors have read and approved the final manuscript. QH is the first author in the list of shared first authors, as she initiated the project and performed all the experiments and data analysis. NT is the second author in the list of shared first authors, as she conducted the majority of the animal experiments. JZ is the third author in the list of shared first authors, as he collected clinical samples for analysis. SS is the fourth author in the list of shared first authors, as she performed data analysis for the ChIP-Seq.

## Supplementary Material

Supplemental data

Unedited blot and gel images

Supplemental tables 1-6

Supporting data values

## Version 1 10/24/2024

In-Press Preview

## Version 2 12/02/2024

Electronic publication
