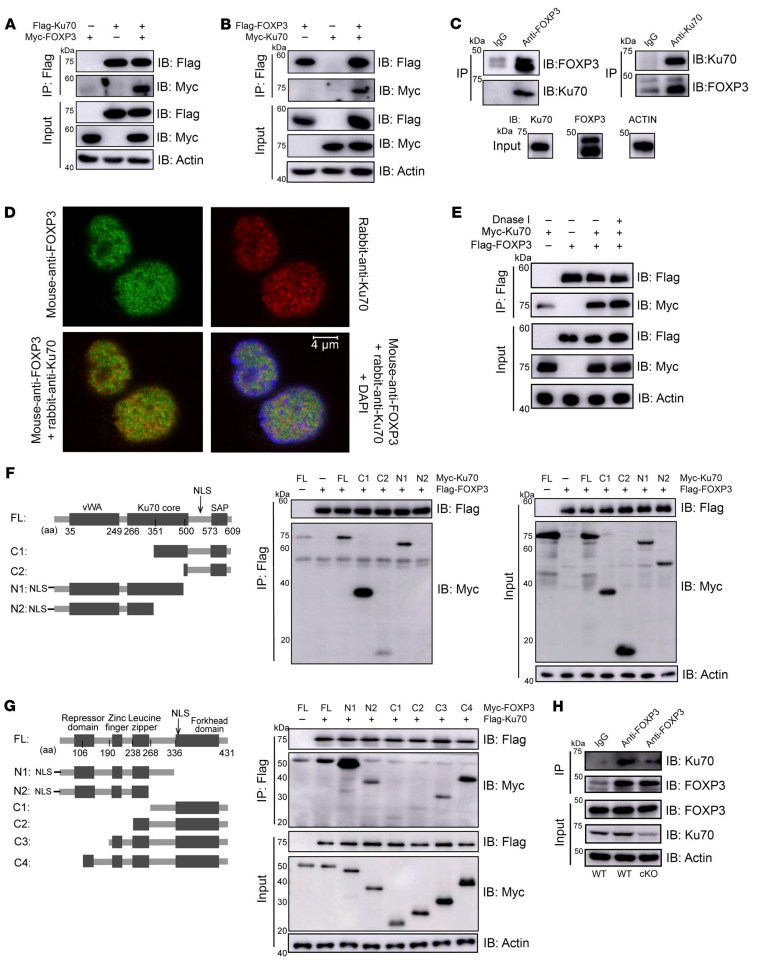# Nonclassical action of Ku70 promotes Treg-suppressive function through a FOXP3-dependent mechanism in lung adenocarcinoma

**DOI:** 10.1172/JCI191305

**Published:** 2025-02-17

**Authors:** Qianru Huang, Na Tian, Jianfeng Zhang, Shiyang Song, Hao Cheng, Xinnan Liu, Wenle Zhang, Youqiong Ye, Yanhua Du, Xueyu Dai, Rui Liang, Dan Li, Sheng-Ming Dai, Chuan Wang, Zhi Chen, Qianjun Zhou, Bin Li

Original citation: *J Clin Invest*. 2024;134(23):e178079. https://doi.org/10.1172/JCI178079

Citation for this corrigendum: *J Clin Invest*. 2025;135(4):e191305. https://doi.org/10.1172/JCI191305

In Figure 6E, the labeling for transfected cells was incorrect. In addition, Figure 6H was not labeled. The correct figure is shown below. The HTML and PDF versions of the paper have been updated.

The authors regret the errors.

## Figures and Tables

**Figure:**