# Supplementary material for: Nonclassical action of Ku70 promotes Treg-suppressive function through a FOXP3-dependent mechanism in lung adenocarcinoma
Source: J Clin Invest. 2024 Oct 24;134(23):e178079. doi: 10.1172/JCI178079 (PMC11601948; doi:10.1172/JCI178079)
Supplement: Supplemental data [file jci-134-178079-s008.pdf]

Supplemental Figure

Figure S1

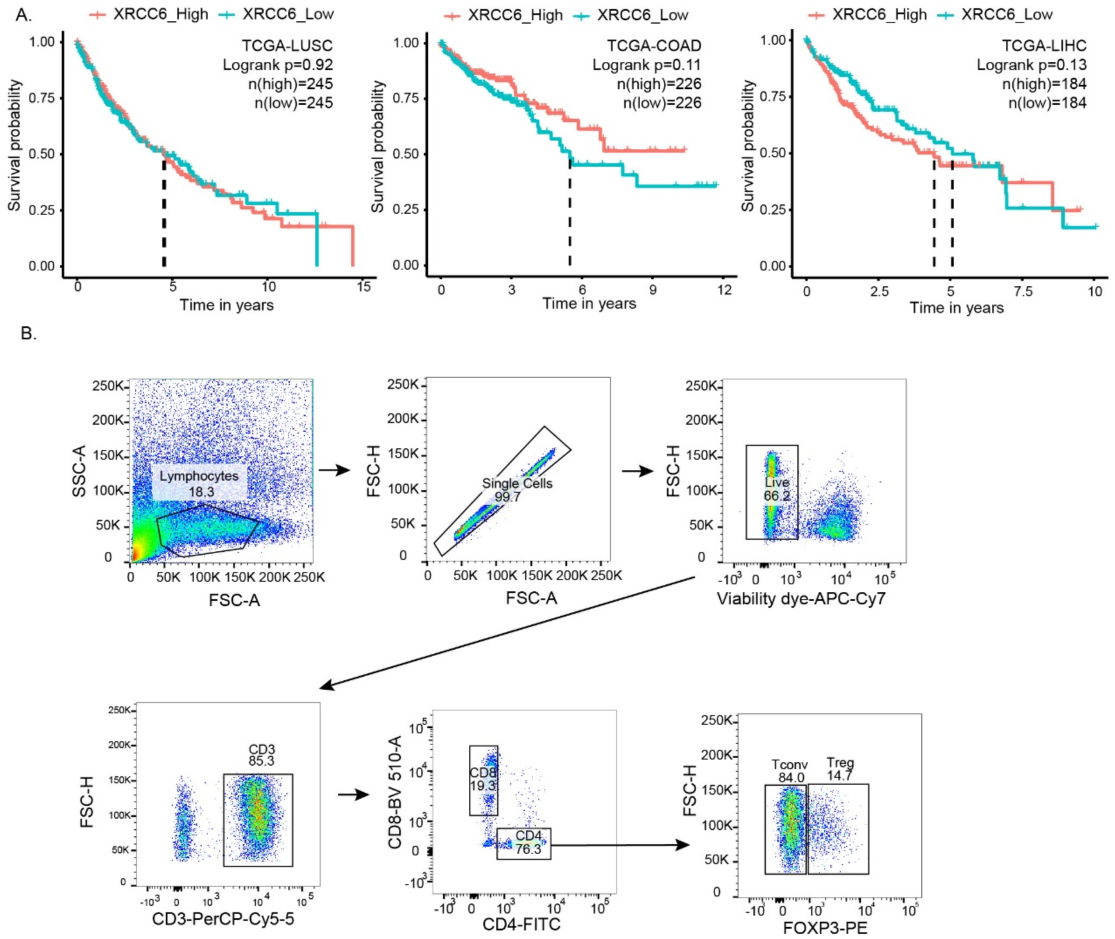

Figure S1 The Kaplan-Meier curves of Ku70 in diverse cancer types and flow cytometry gating strategy of patient samples. (A) Overall survival analysis based on *XRCC6* gene expression (median) using Kaplan–Meier curves in the TCGA-LUSC, TCGA-COAD and TCGA-LIHC cohort. The red line represents patients with high *XRCC6* expression, while the blue line represents those with low expression. P-values were calculated using log-rank test. (B) Flow cytometry gating strategy of the patient samples to isolate CD8+ T cells, $CD4^{+}FOXP3^{-}$ Tconv cells and $CD4^{+}FOXP3^{+}$ Treg cells.

Figure S2

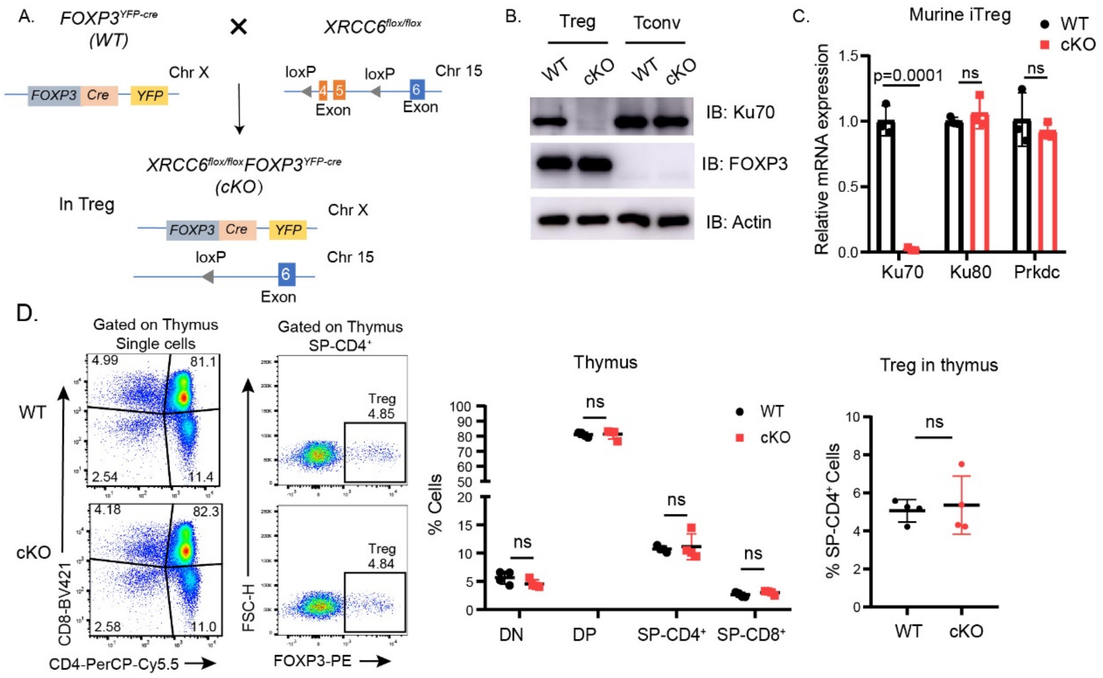

Figure S2 The development of T cell was comparable in WT and cKO mice. (A) Schematic illustration of the strategy used to generate Treg-specific Ku70 knockout mice. (B) Immunoblot analysis of Ku70 expression in Treg cells and Tconv cells from WT and cKO mice. (C) qRT-PCR analysis of Ku70, Ku80 and Prkdc expression in Treg cells and Tconv cells from WT and cKO mice. (D) $CD4^-CD8^-$ double negative (DN), $CD4^+CD8^+$ double positive (DP), $CD4^+$ T cells (SP-$CD4^+$), $CD8^+$ T cells (SP-$CD8^+$) and Treg cells, in the thymus from WT and cKO mice (6-week-old, n=4). Data are representative of 3 independent experiments. Data are represented as the mean ± SD. The significances were measured by unpaired two-tailed Student's *t*-test. NS, not significant.

Figure S3

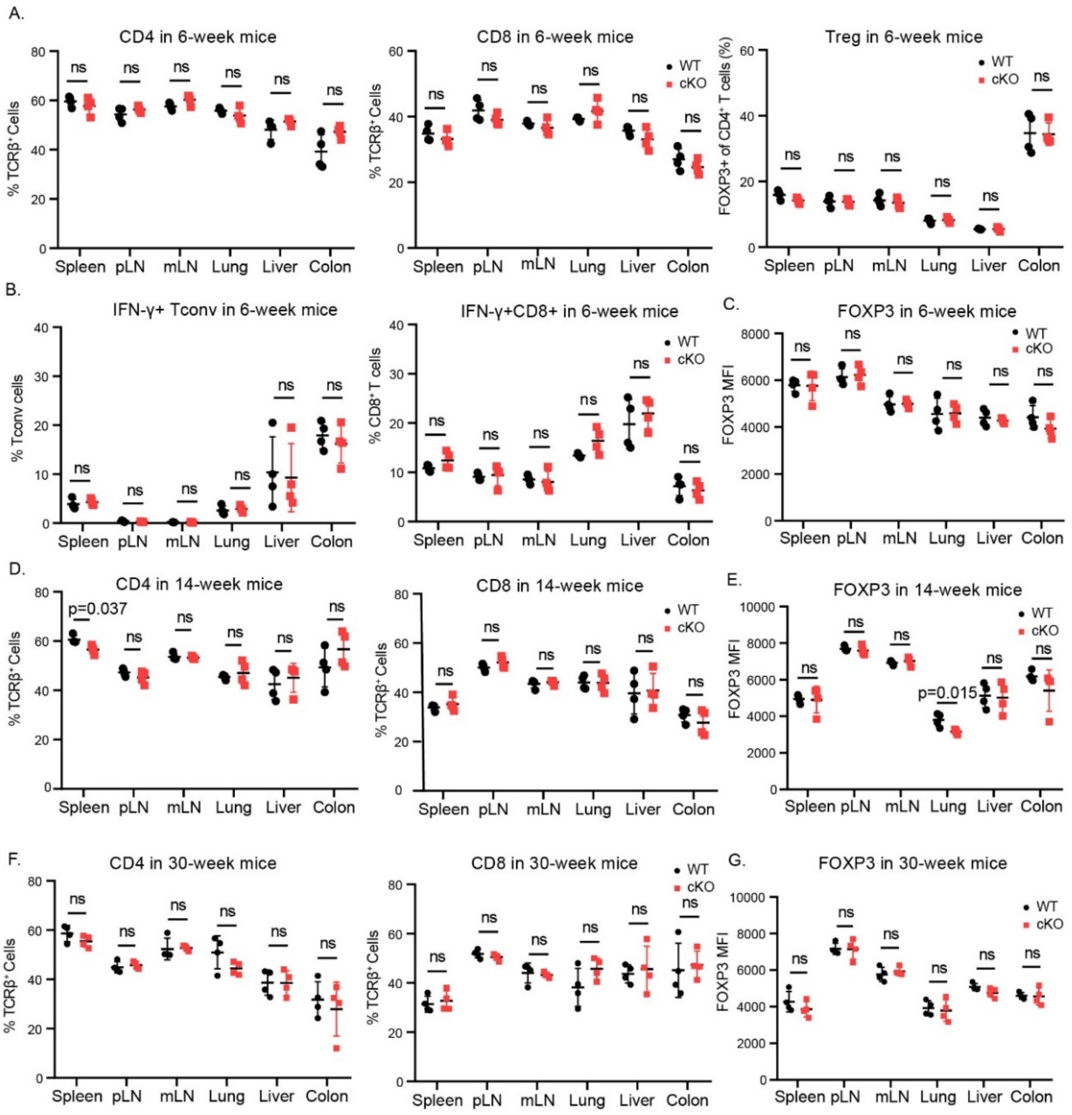

Figure S3. Alterations in immune homeostasis in various tissues of mice at different ages. (A) Frequency of $CD4^+$, $CD8^+$ T and Treg cells in different organs in WT and cKO mice (6-week-old, n=4). (B) Frequency of IFN-γ producing Tconv and $CD8^+$ T cells in different organs in WT and cKO mice (6-week-old, n=4). (C) Mean fluorescence intensity of FOXP3 in different organs in WT and cKO mice (6-week-old, n=4). (D) Frequency of $CD4^+$, $CD8^+$ T and Treg cells in different organs in WT and cKO mice (14-week-old, n=4). (E) Mean fluorescence intensity of FOXP3 in different organs in WT and cKO mice (14-week-old, n=4). (F) Frequency of $CD4^+$, $CD8^+$ T and Treg cells in different organs in WT and cKO mice (30-week-old, n=4). (G) Mean fluorescence intensity of FOXP3 in different organs in WT and cKO mice (30-week-old, n=4). Data are represented as the mean ± SD. The significances were measured by unpaired two-tailed Student’s t-test. ns, not significant.

Figure S4

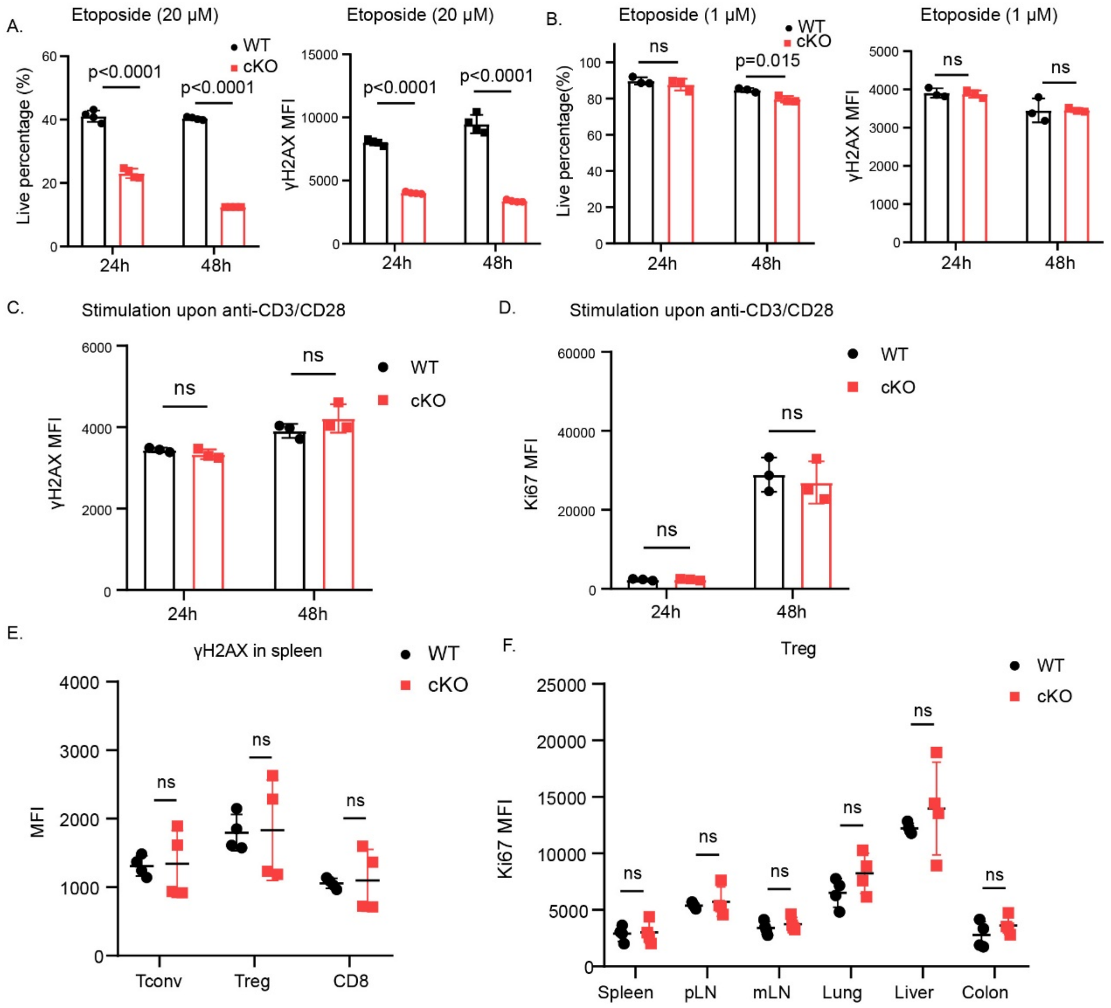

Figure S4 Changes in DNA damage and proliferation status in Ku70-deficient Treg cells. (A-B) Natural Treg cells were sorted from the spleens of WT or cKO mice (8-week-old) and then treated with the topoisomerase II inhibitor etoposide for 24 or 48 hours in vitro. The cells were collected to assess cell viability and the expression of the DNA damage marker γH2AX by flow cytometry. (C-D) Natural Treg cells were sorted from the spleens of WT or cKO mice (8-week-old) and then stimulated with anti-CD3/CD28 beads for 24 or 48 hours in vitro. The cells were collected and analyzed by flow cytometry for the expression of DNA damage marker γH2AX and cell proliferation marker Ki67. (E-F) The expression of proliferation marker Ki67 and DNA damage marker γH2AX in T cells from WT and cKO mice (14-week-old, n=4). Data are represented as the mean ± SD. The significances were measured by unpaired two-tailed Student's t-test. ns, not significant.

Figure S5

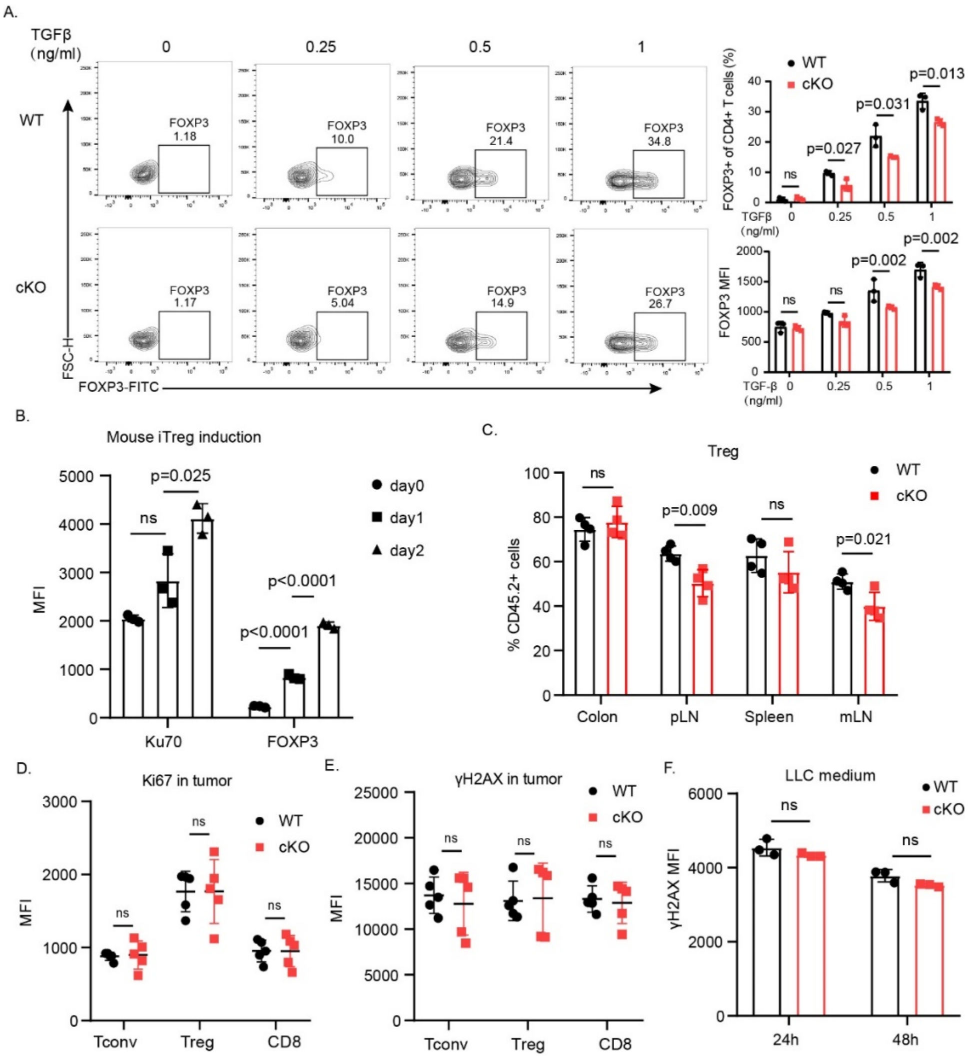

Figure S5 Ku70 affects the induced differentiation of Treg cells. (A) Naïve CD4$^+$ T cells were sorted from spleen of WT and cKO mice (14-week-old), then, were induced into iTreg cells in vitro. The differentiation efficiency of Tregs was determined by the frequencies of Foxp3$^+$ cells. (B) Naïve CD4$^+$ T cells were sorted from spleen of WT mice (8-week-old), then, were induced into iTreg cells in vitro. The expression levels of FOXP3 and Ku70 were assessed by flow cytometry. (C) Frequency of CD45.2$^+$ Treg cells in different organs in recipient mice (n=4) with adoptive transfer colitis models at six weeks. (D-E) The expression of proliferation marker Ki67 and DNA damage marker γH2AX in tumor-infiltrating T cells from B16F10-bearing WT and cKO mice (n=5). (F) Natural Treg cells were sorted from the spleens of WT or cKO mice (8-week-old) and then treated with LLC conditional medium for 24 or 48 hours in vitro. The cells were collected to assess the expression of the DNA damage marker γH2AX by flow cytometry. Data are represented as the mean ± SD. The significances were measured by unpaired two-tailed Student's *t*-test. NS, not significant.

Figure S6

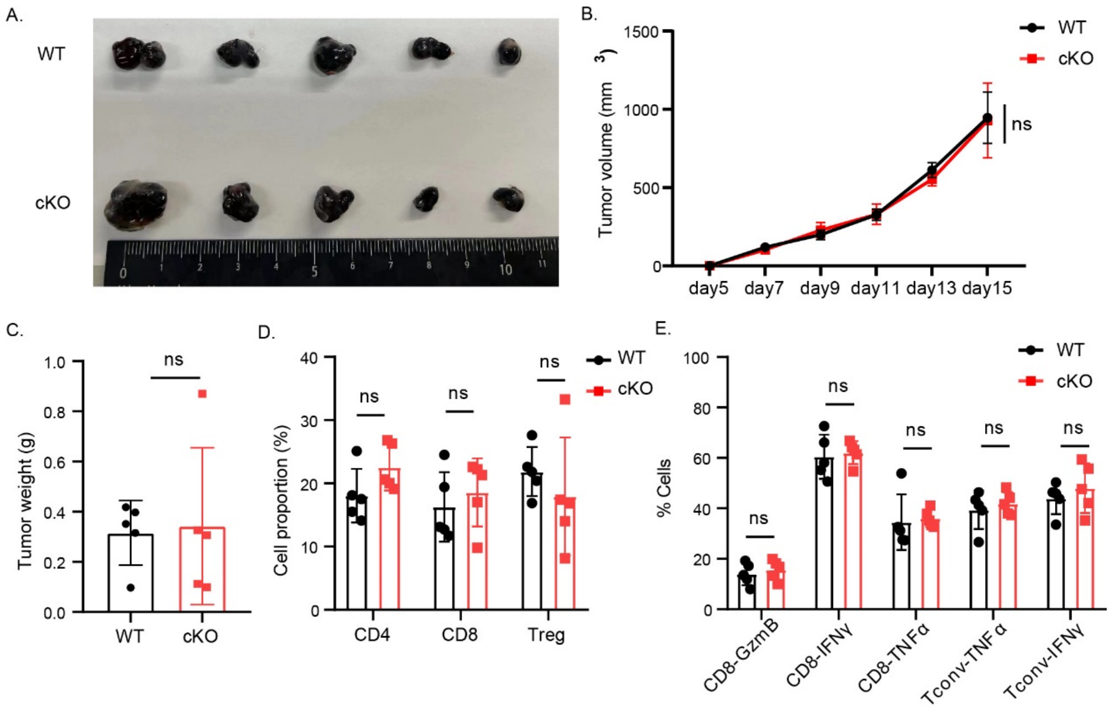

Figure S6 The deletion of Ku70 in Treg cells fails to inhibit the growth of the B16F10 subcutaneous tumor model. (A) Macroscopic evaluation of subcutaneous tumor size from B16-bearing WT and cKO mice (n=5) on day 15 post-injection. (B) Tumor growth curves are summarized. Data are represented as the mean ± SEM. (C) The final tumor volumes after 15 days are summarized. (D) Frequencies of $CD4^+$, $CD8^+$ T and Treg cells in B16 subcutaneous tumors from WT and cKO mice (n=5). (E) Frequency of GzmB, IFNγ and TNFα producing $CD4^+$ and $CD8^+$ T cells in B16 subcutaneous tumors of WT and cKO mice (n=5). Data are represented as the mean ± SD. The significances were measured by unpaired two-tailed Student's t-test. ns, not significant.

Figure S7

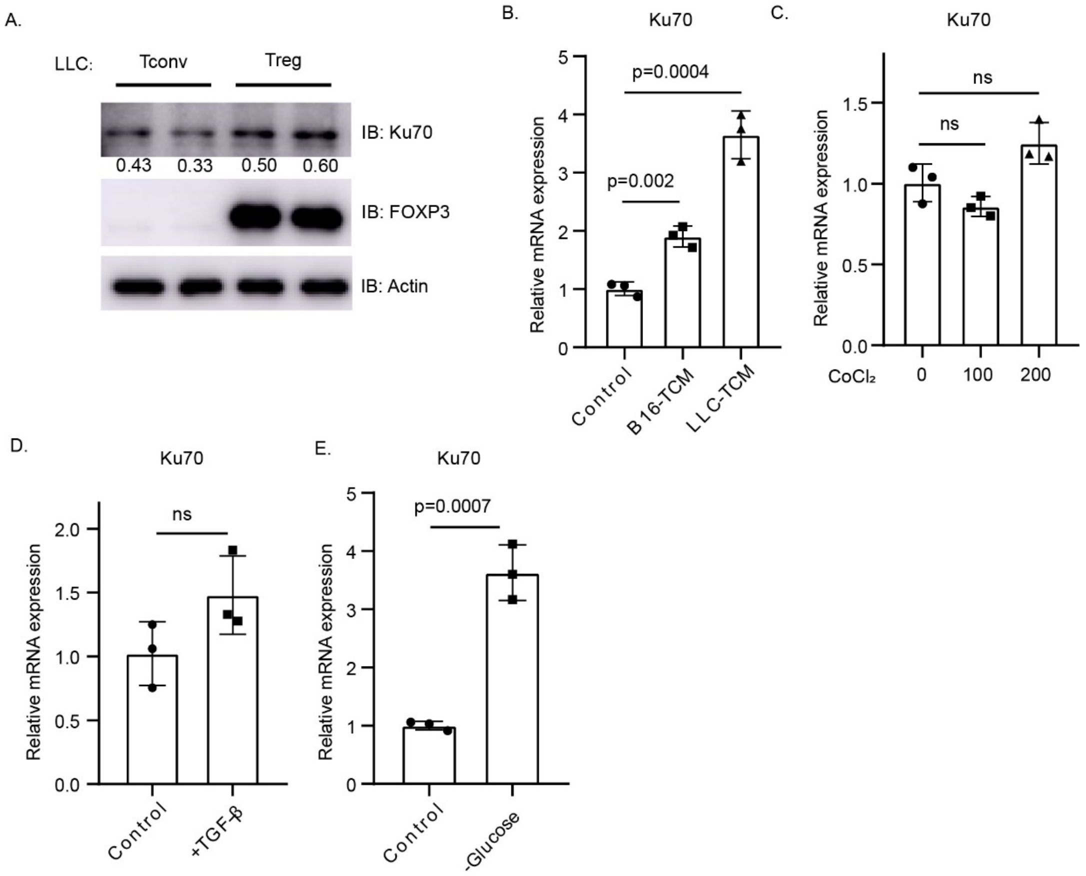

Figure S7 The impact of the tumor microenvironment on Ku70 expression in Treg cells. (A) The detection of Ku70 expression in different T-cell types from murine tumor models by immunoblotting assay. These cells were isolated from LLC tumor-bearing mice at 21 days after tumor injection by flow cytometry. (B) Ku70 mRNA level in human iTreg cells in control T cell medium compared to addition of tumor cell–treated medium at 50:50 with T cell medium for 24 hours (n=3). (C) The mRNA level of Ku70 in human iTreg cells was measured after treatment with different concentrations of $CoCl_2$ (0, 100, 200μM) for 24 hours to induce a hypoxic environment (n=3). (D) The mRNA level of Ku70 in human iTreg cells with the addition of TGF-β (16ng/ml) in medium for 24 hours (n=3). (E) The mRNA level of Ku70 in Treg cells after exposure to glucose-restricted conditions after 24 hours relative to normal medium (n=3). Data are represented as the mean ± SD. The significances were measured by unpaired two-tailed Student's t-test. ns, not significant.

Figure S8

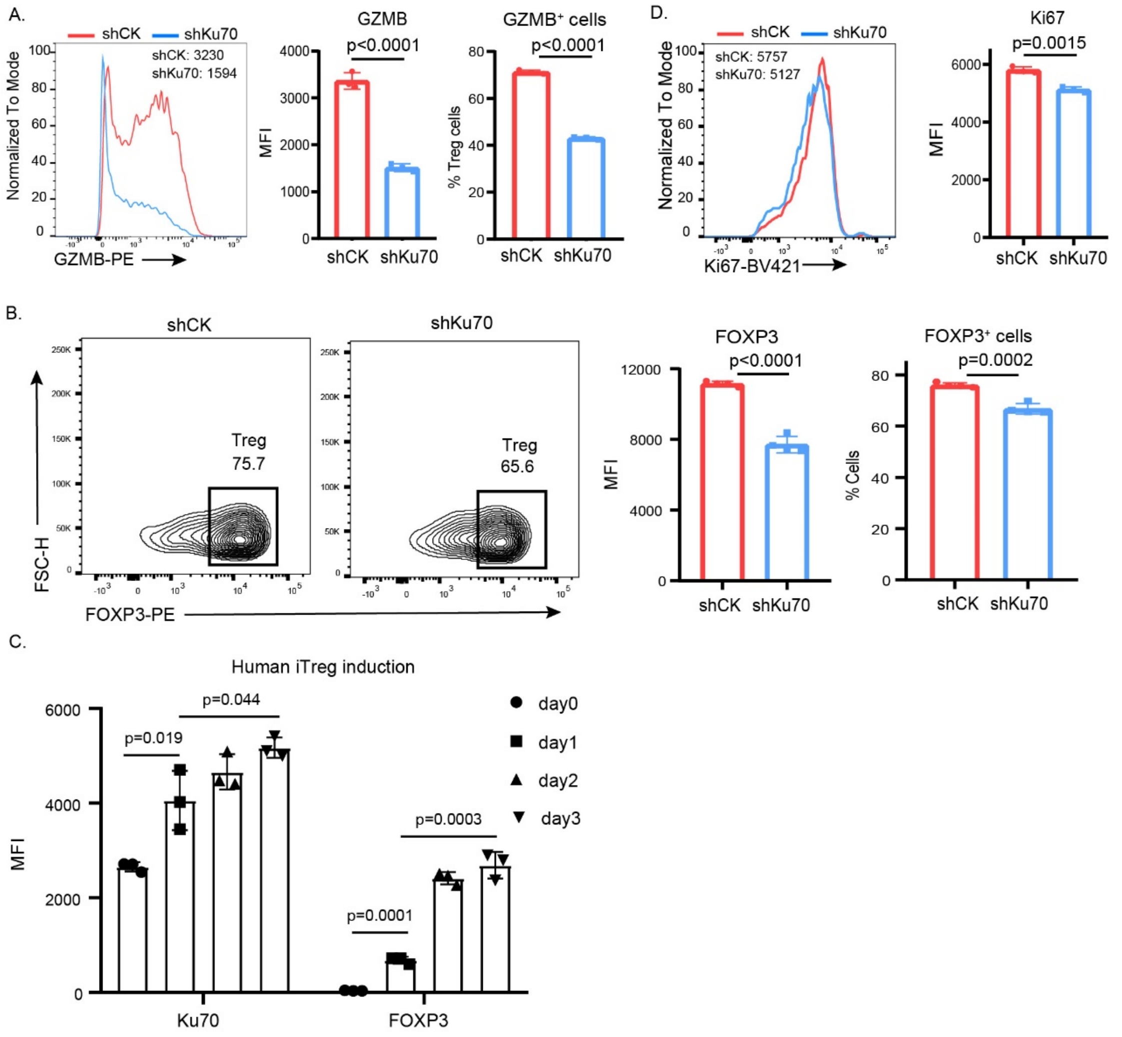

Figure S8 The effect of Ku70 knockdown on human iTreg cells. (A) GZMB production from human iTreg cells were assessed after knockdown of Ku70 by lentiviruses carrying Ku70 shRNA. (B) Ku70 was knocked down in naïve CD4$^+$ T cells by shKu70 lentivirus. After puromycin selection, the human naïve CD4$^+$ T cells with Ku70 knockdown were induced into iTreg cells in vitro. The differentiation efficiency of Tregs was determined by the frequencies of FOXP3$^+$ cells. (C) Naïve CD4$^+$ T cells were sorted from donor PBMC, then, were induced into iTreg cells *in vitro*. The expression levels of FOXP3 and Ku70 were assessed by flow cytometry. (D) Representative histogram and the quantification of Ki67 MFI in Treg cells carrying shCK or shKu70. Data are representative of 3 independent experiments. Data are represented as the mean ± SD. The significances were measured by unpaired two-tailed Student's *t*-test. NS, not significant.

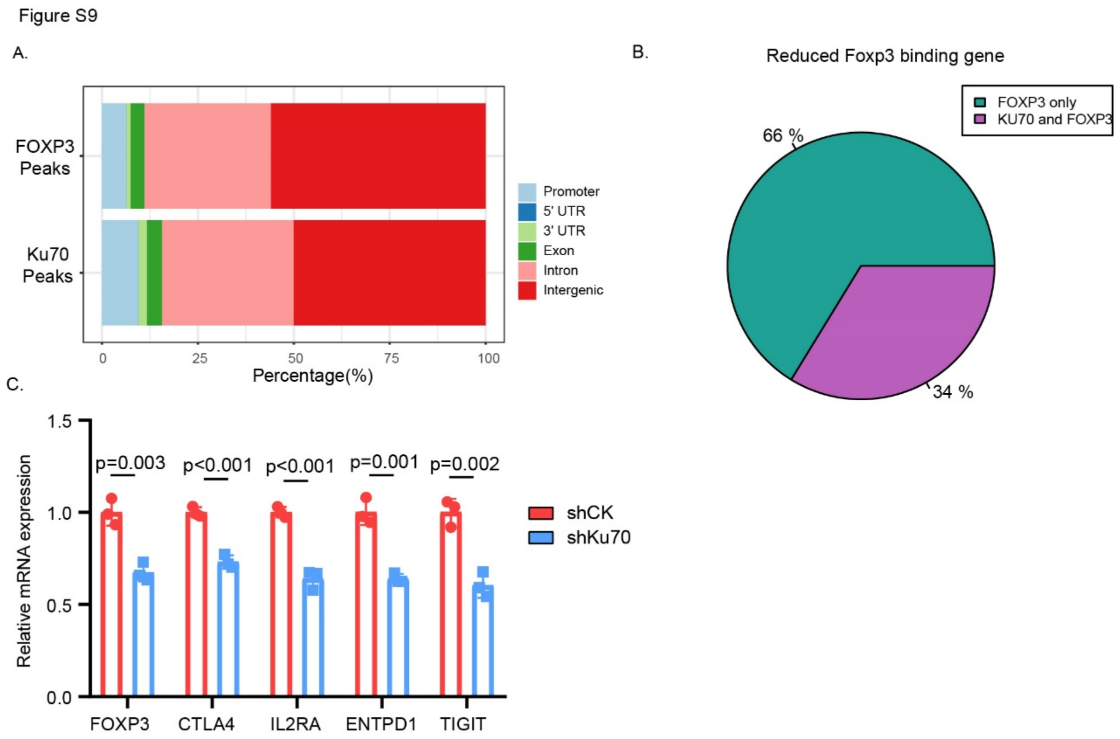

Figure S9. Chip-seq data confirmed that Ku70 supports the activity of FOXP3 transcription. (A) Peak annotation of FOXP3-binding and Ku70-binding sites in Treg cells. (B) Pie chart illustrated the percentage of FOXP3- and Ku70-binding within the reduced FOXP3-binding sites. (C) qRT-PCR analysis of down-regulated genes in Ku70 knockdown human Treg cells.
