# Supplementary material for: Nonclassical action of Ku70 promotes Treg-suppressive function through a FOXP3-dependent mechanism in lung adenocarcinoma
Source: J Clin Invest. 2024 Oct 24;134(23):e178079. doi: 10.1172/JCI178079 (PMC11601948; doi:10.1172/JCI178079)

Full unedited gel for Figure 5A

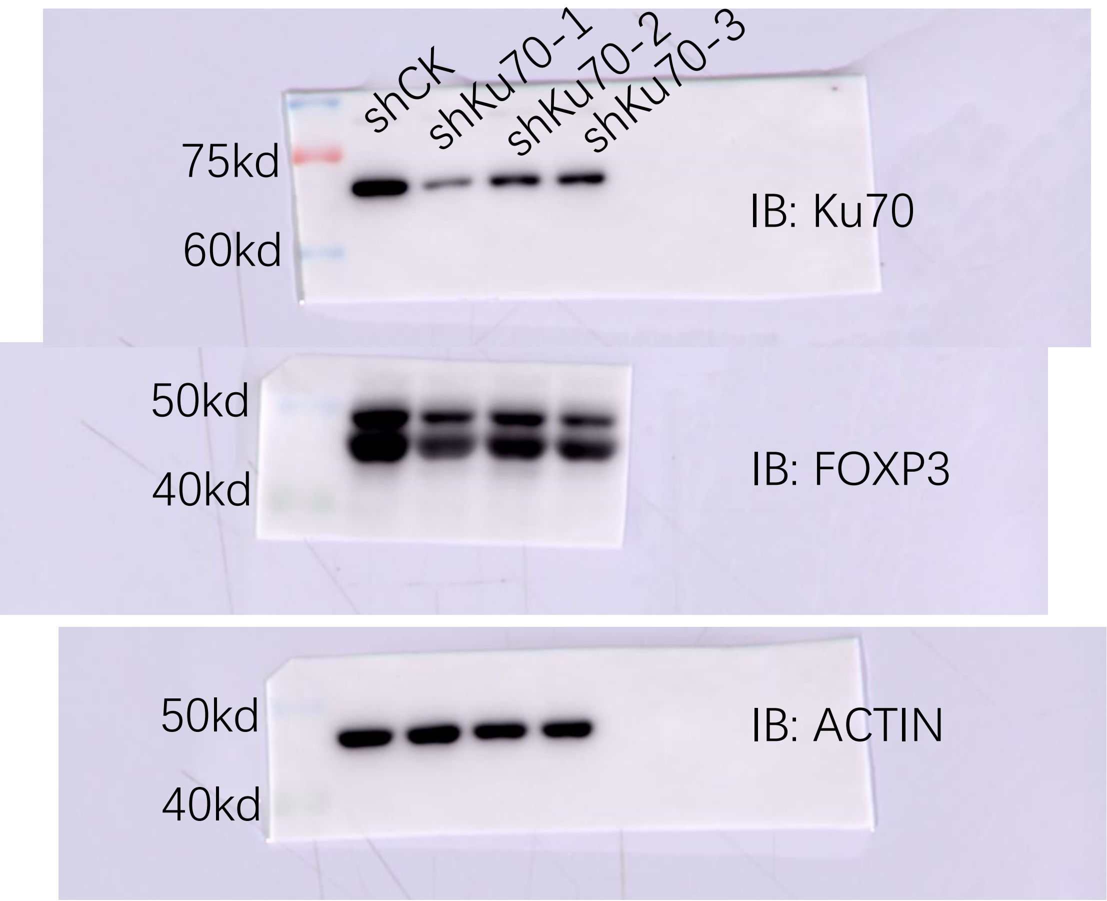

Full unedited gel for Figure 6A

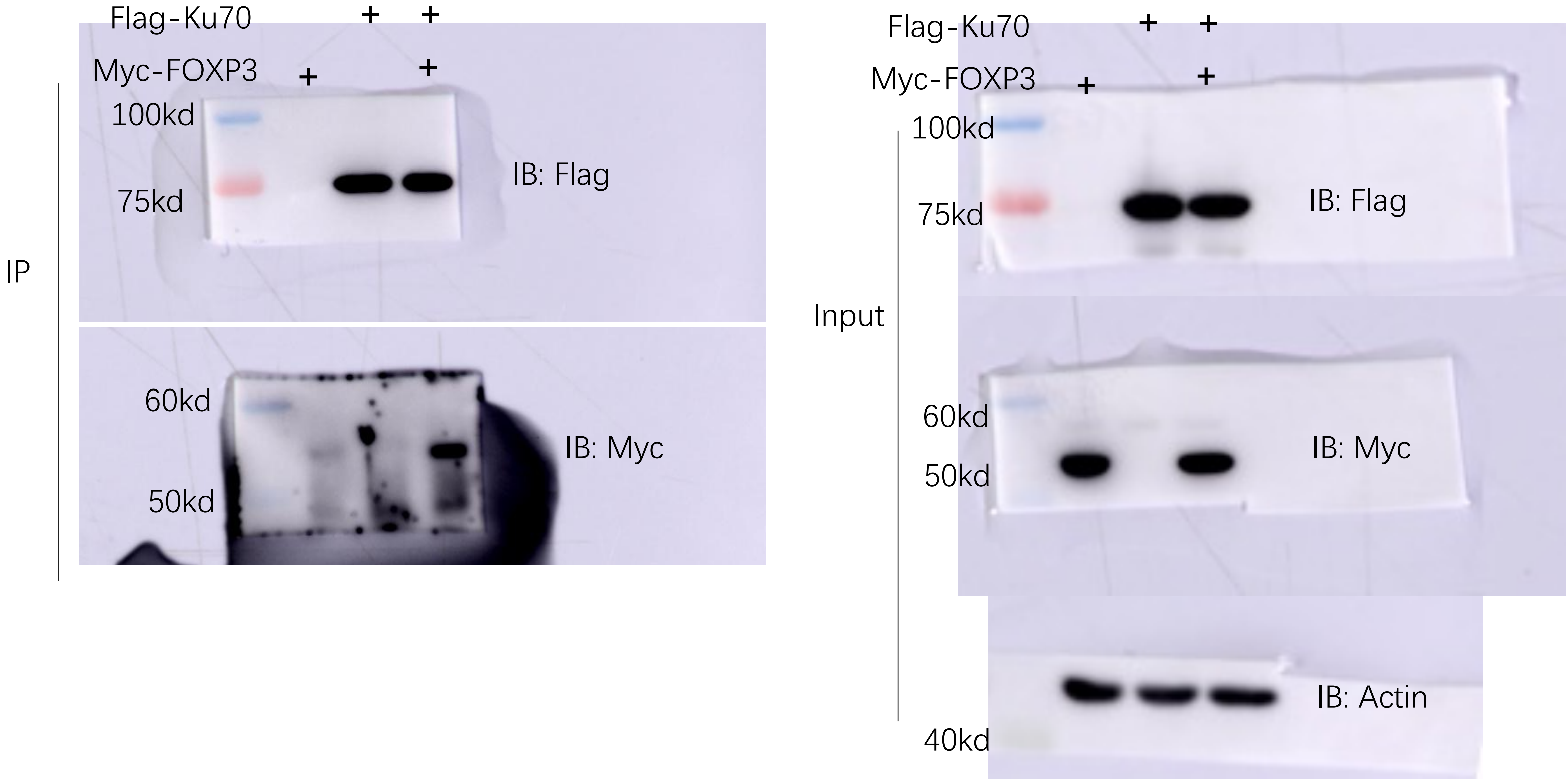

Full unedited gel for Figure 6B

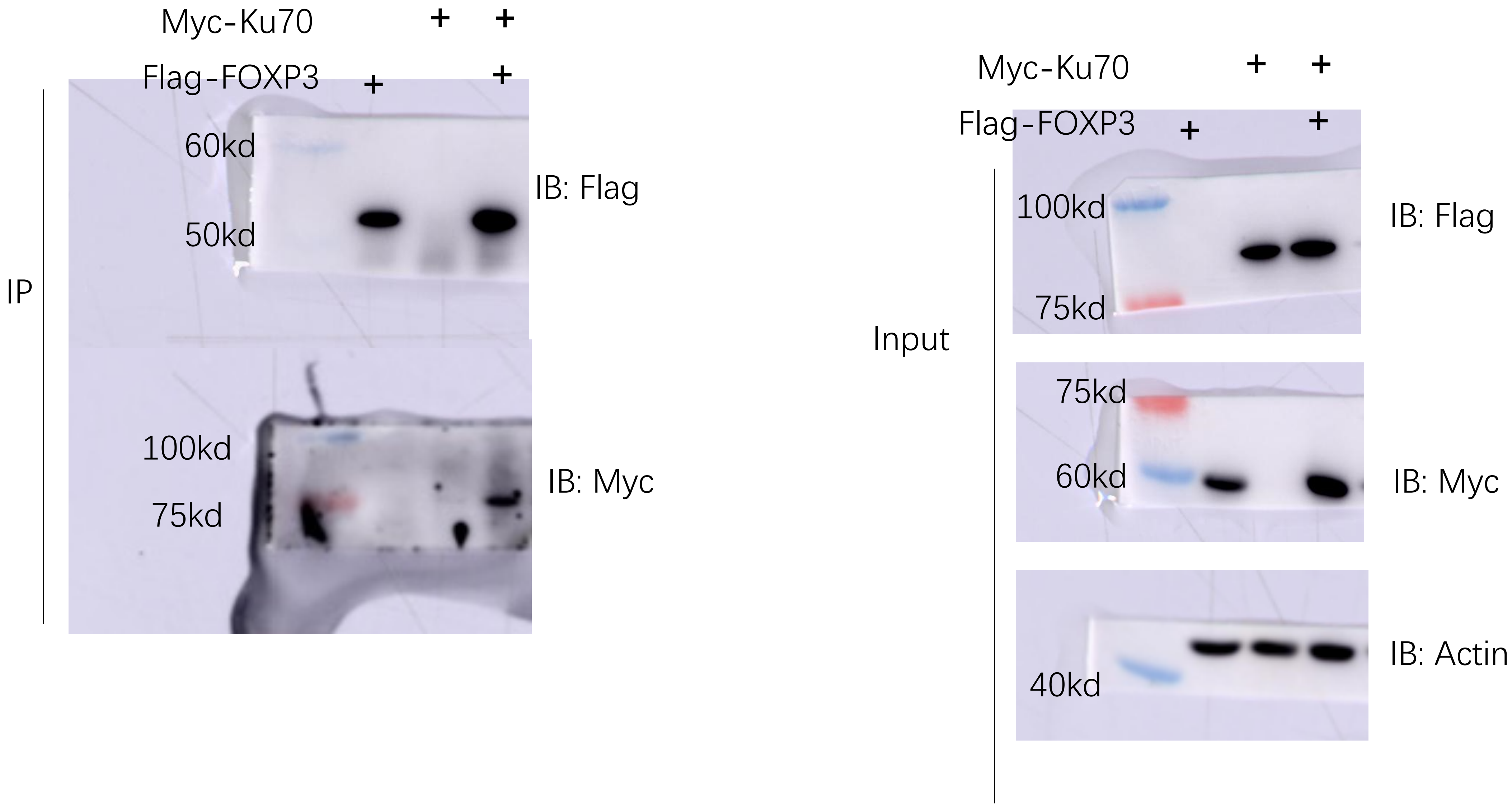

Full unedited gel for Figure 6C

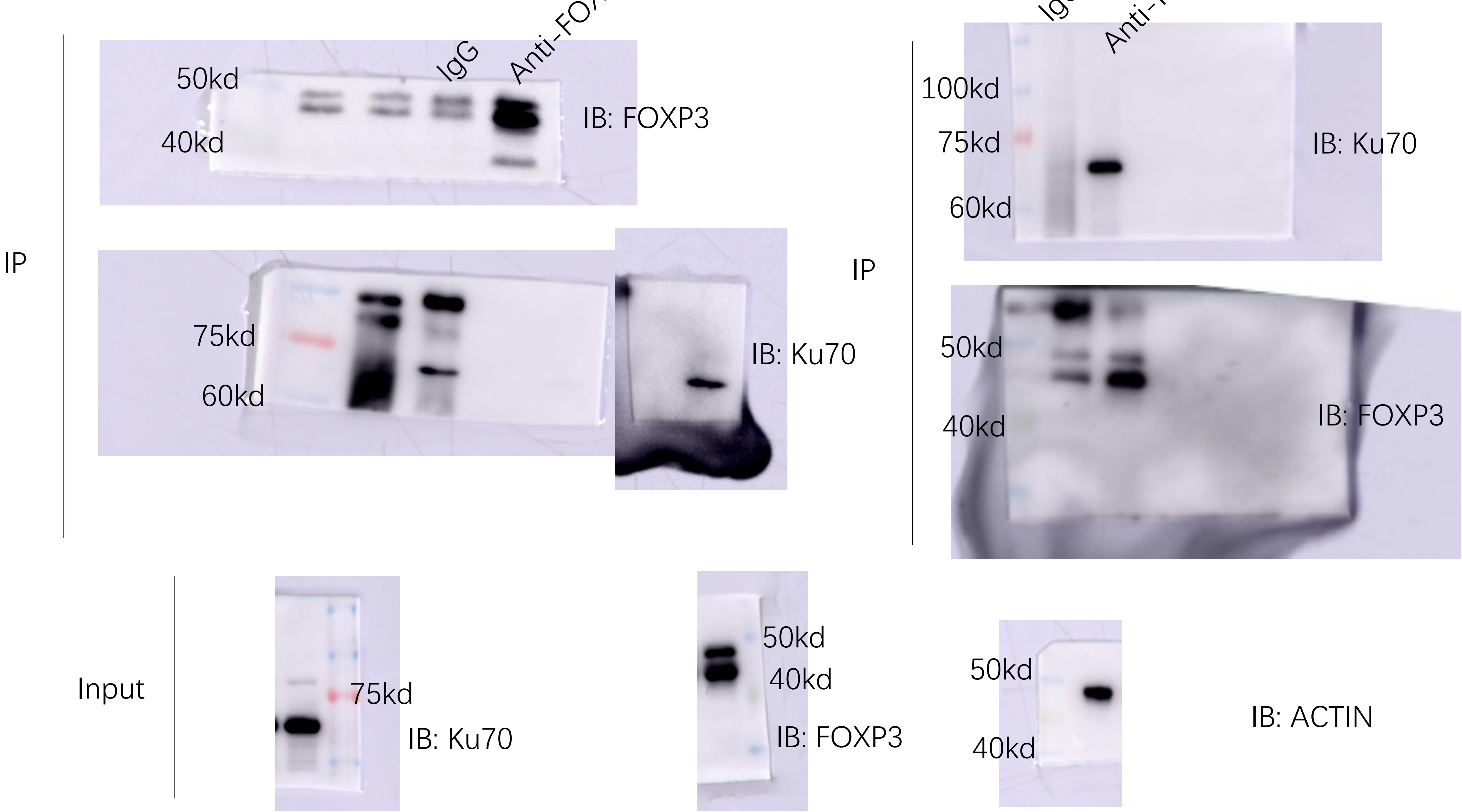

Full unedited gel for Figure 6E

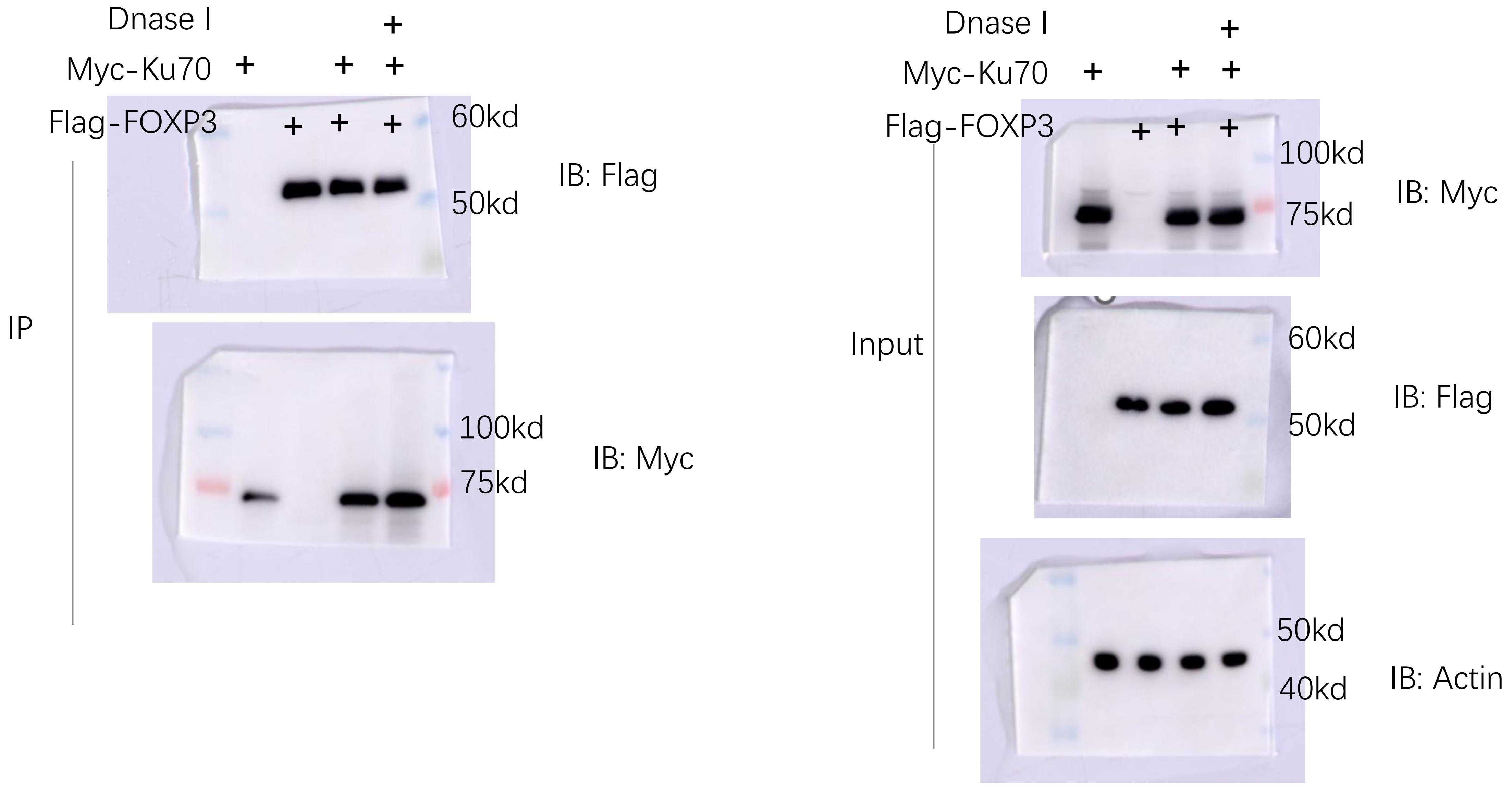

Full unedited gel for Figure 6F

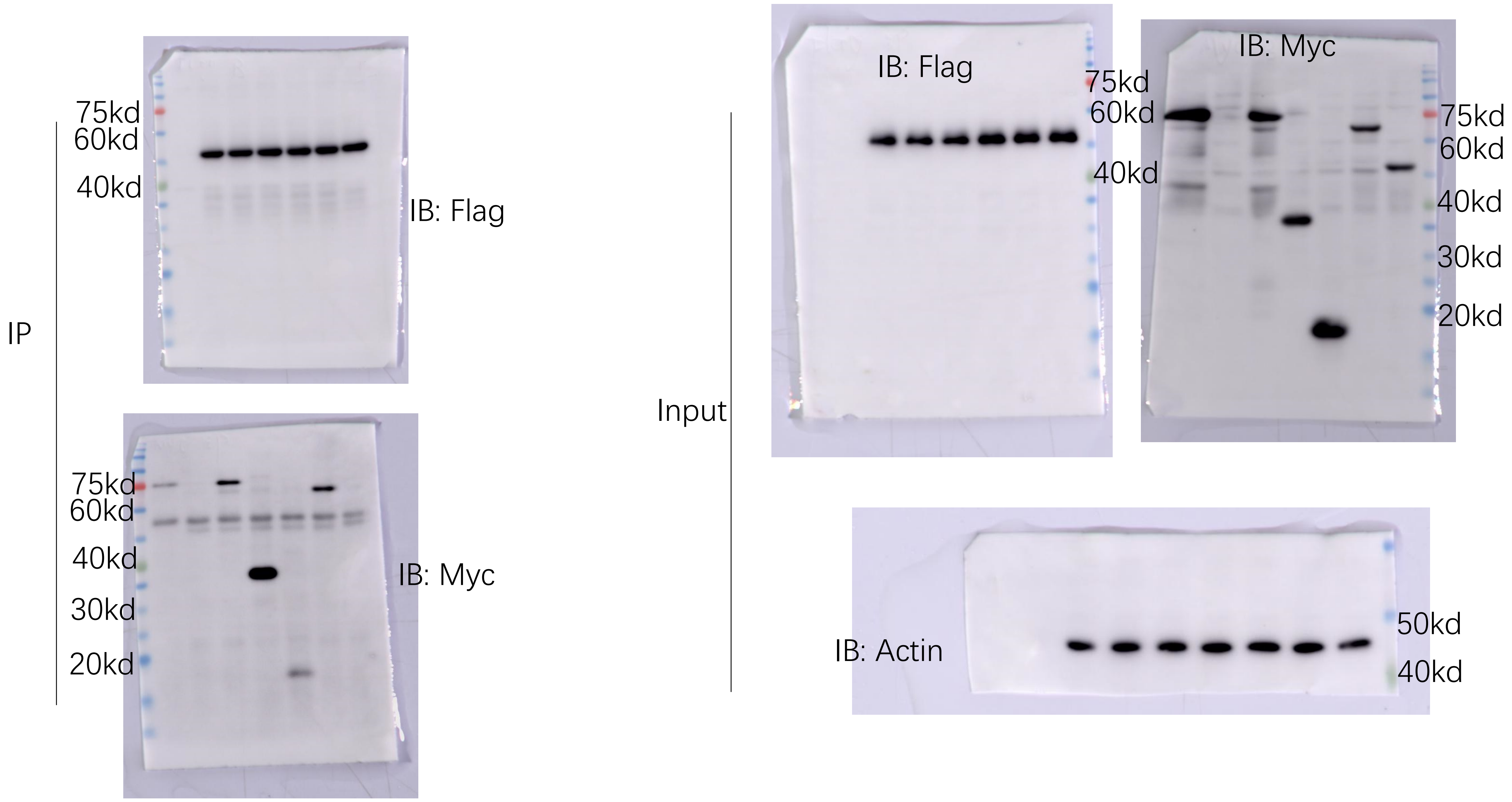

Full unedited gel for Figure 6G

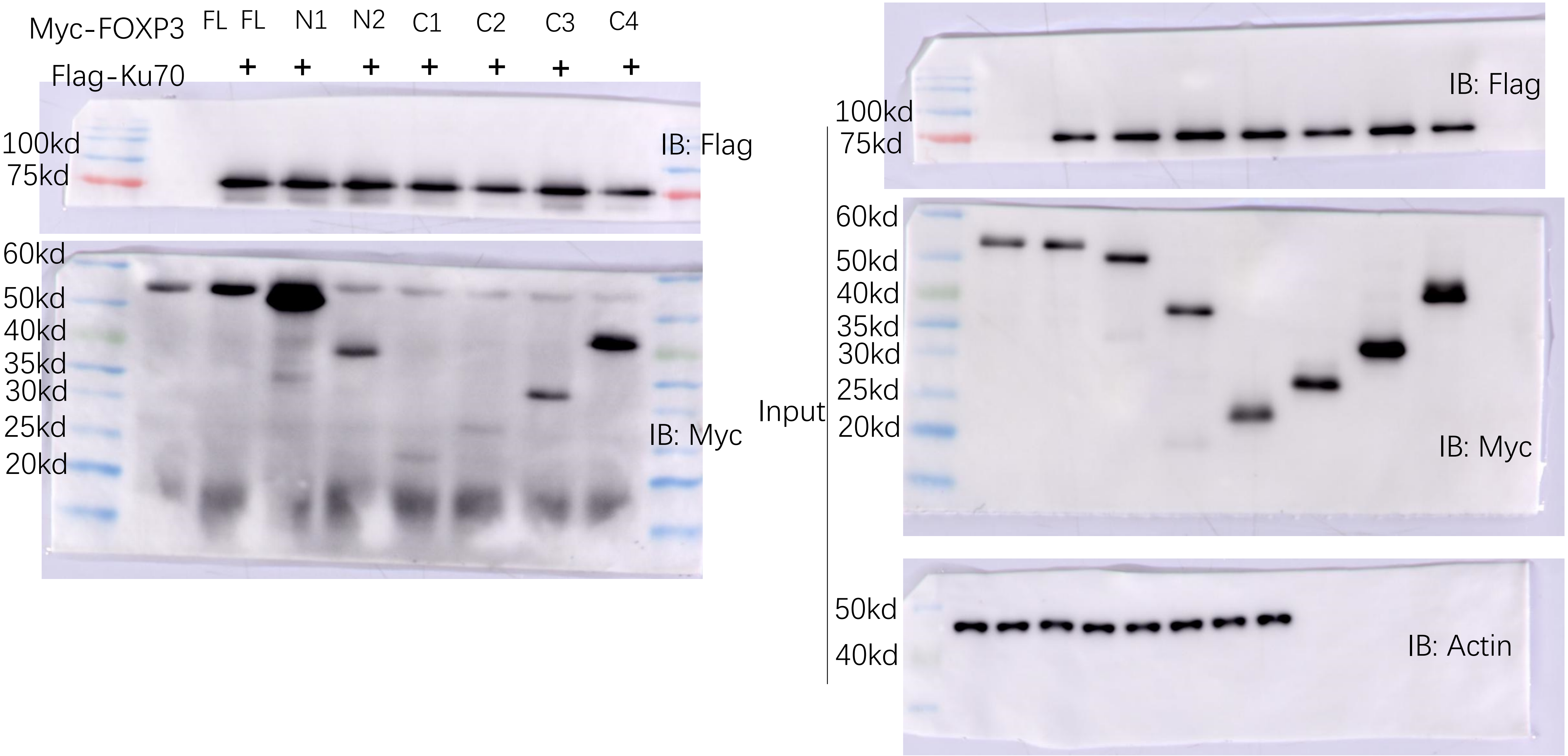

Full unedited gel for Figure 6H

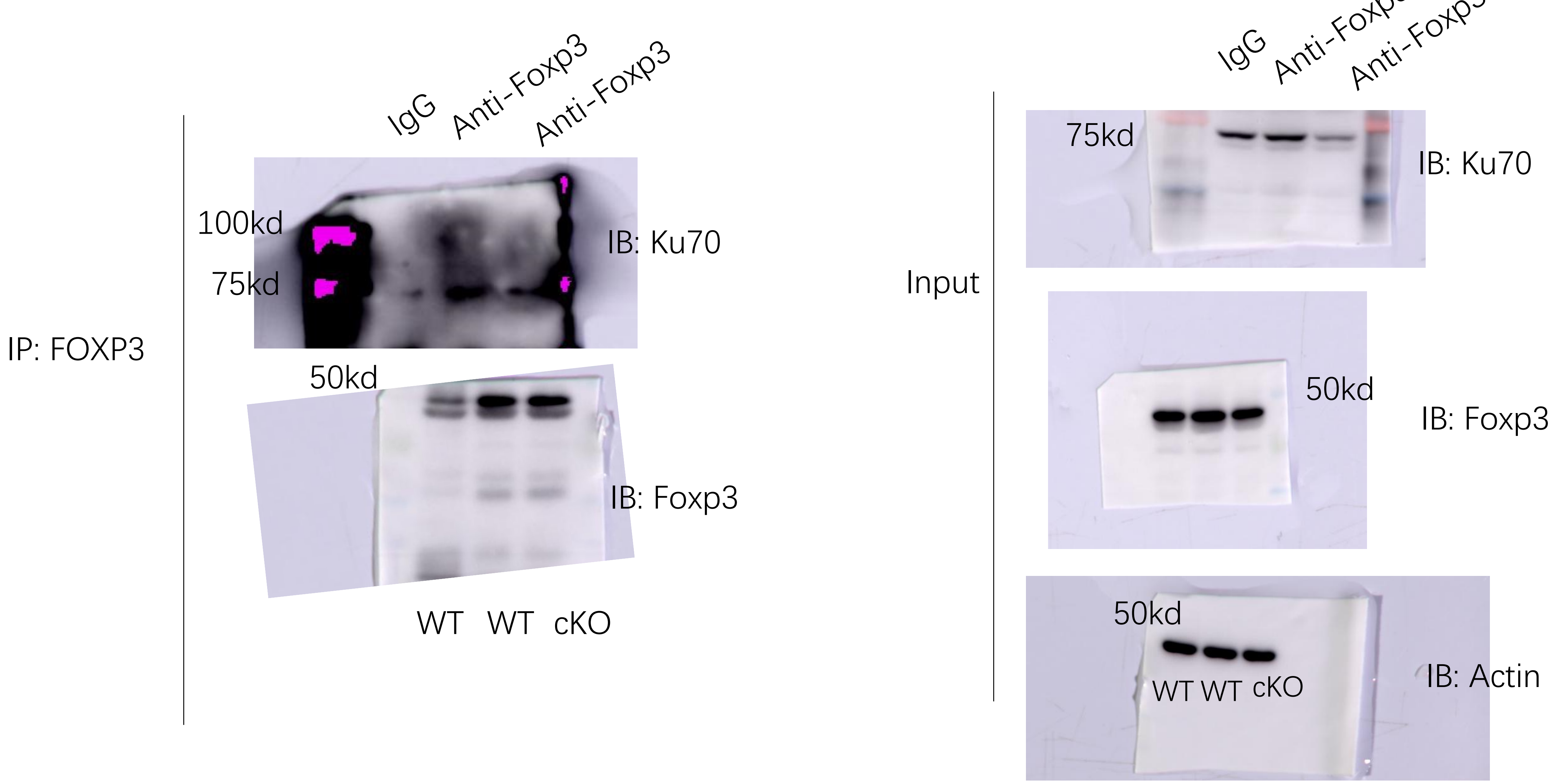

Full unedited gel for supplemental Figure 2B

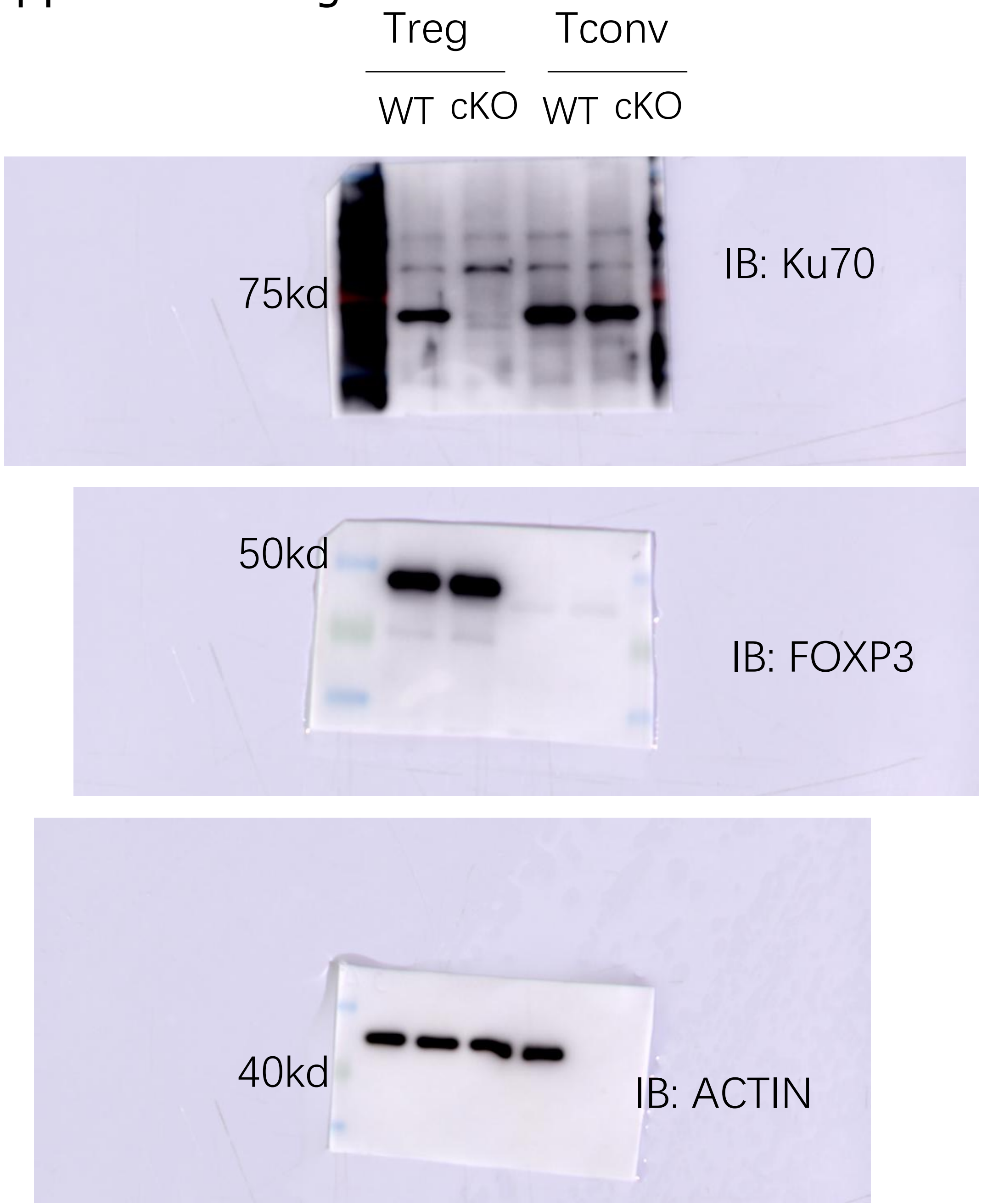

Full unedited gel for supplemental Figure 7A

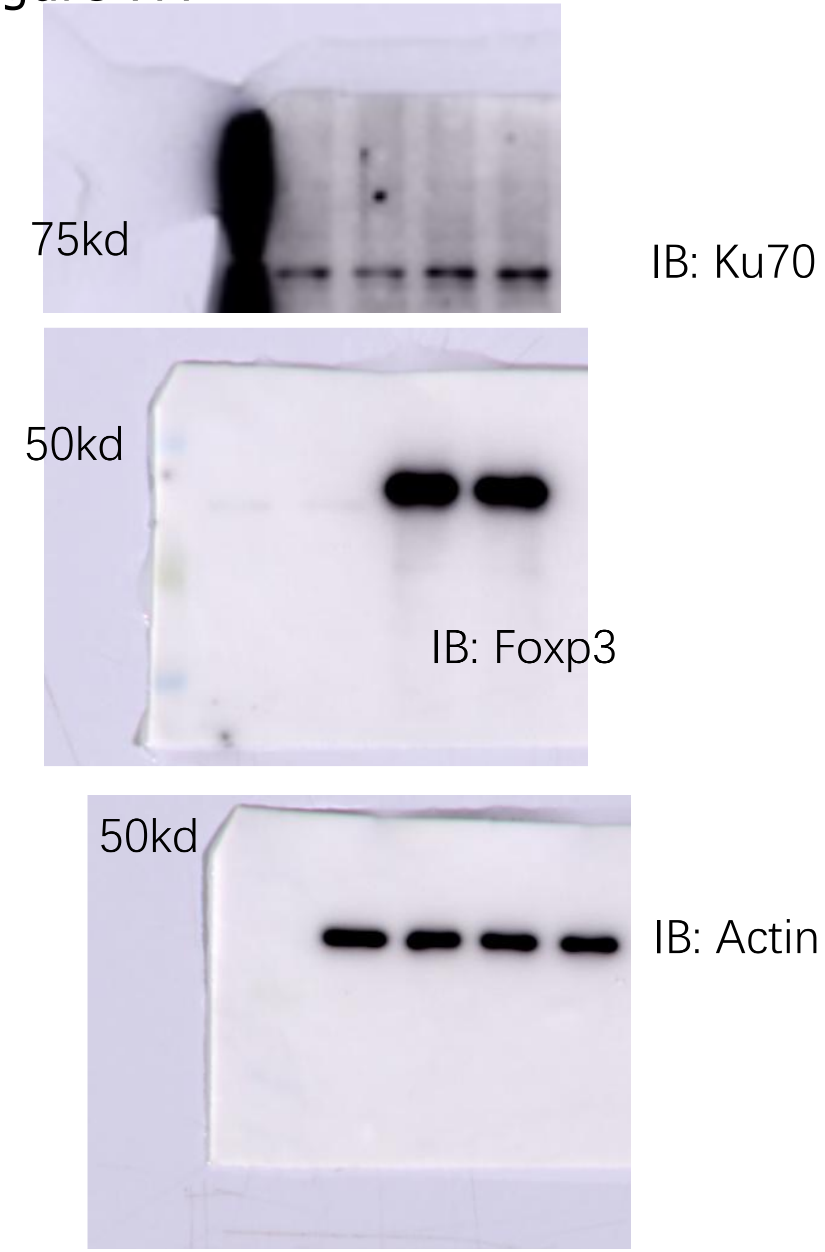

Supplement: Unedited blot and gel images [file jci-134-178079-s009.pdf]
